# Key innovation or adaptive change? A test of leaf traits using Triodiinae in Australia

**DOI:** 10.1038/srep12398

**Published:** 2015-07-28

**Authors:** A. Toon, M. D. Crisp, H. Gamage, J. Mant, D. C. Morris, S. Schmidt, L. G. Cook

**Affiliations:** 1 School of Biological Sciences, The University of Queensland, Brisbane QLD 4072, Australia; 2 Research School of Biology, The Australian National University, Canberra ACT 2601, Australia; 3 School of Agriculture and Food Sciences, The University of Queensland, Brisbane QLD 4072, Australia

## Abstract

The evolution of novel traits (“key innovations”) allows some lineages to move into new environments or adapt to changing climates, whereas other lineages may track suitable habitat or go extinct. We test whether, and how, trait shifts are linked to environmental change using Triodiinae, C_4_ grasses that form the dominant understory over about 30% of Australia. Using phylogenetic and relaxed molecular clock estimates, we assess the Australian biogeographic origins of Triodiinae and reconstruct the evolution of stomatal and vascular bundle positioning. Triodiinae diversified from the mid-Miocene, coincident with the aridification of Australia. Subsequent niche shifts have been mostly from the Eremaean biome to the savannah, coincident with the expansion of the latter. Biome shifts are correlated with changes in leaf anatomy and radiations within Triodiinae are largely regional. *Symplectrodia* and *Monodia* are nested within *Triodia*. Rather than enabling biome shifts, convergent changes in leaf anatomy have probably occurred after taxa moved into the savannah biome—they are likely to have been subsequent adaptions rather than key innovations. Our study highlights the importance of testing the timing and origin of traits assumed to be phenotypic innovations that enabled ecological shifts.

It is expected that the evolution of novel traits allows some lineages to adapt to changing environments while other lineages remain restricted to environments similar to that of their ancestors (e.g.)^1^. Examples of such “key innovations” include the evolution of C_4_ grasses in concert with decreasing atmospheric CO_2_^2^ and the evolution of specialised structural plant cells (wide-band tracheids) in arid environments^3^. However, some traits that appear linked to ecological shifts might be adaptations that occurred subsequent to the move into novel environments, such as the repeated changes in armament of sticklebacks after moving from marine to fresh water habitats^4^. In plants, leaf-related traits are often inferred to be key functional traits allowing adaptation to different environments. There is a clear temperature-related pattern in leaf size among closely related plants (e.g.)^5^, and cladodes and stem succulence have evolved multiple times in taxa of the arid zones (e.g.)^6^. Here we test the direction and order of origin of traits that have been hypothesised to be related to niche shifts in the Australian C_4_ grasses of subtribe Triodiinae (tribe Cynodonteae)^7^, which form the dominant understory over about 30% of Australia^8^,^9^, primarily in the arid and savannah biomes.

Species of Triodiinae are commonly called “spinifex” or “porcupine grass” because of their needle-like leaves. In some species, stomata do not occur on the outer (abaxial) surface and are restricted to the adaxial surface that is protected within the rolled leaf (Fig. 1). The loss of stomata on the abaxial surface in Triodiinae has been hypothesised to be a response to increased aridity^10^, although Mant *et al.*^11^ argued that it was related to shifts into the savannah biome. Here, we test whether loss of abaxial stomata was a key innovation that allowed Triodiinae to shift in ecological niche space during the drying climates of the Cenozoic.

Global cooling over the past 50 Myr has led to major expansions in dry-open habitats worldwide, and extinction of mesic-adapted taxa that have failed to adapt to the drier conditions (e.g.)^12^,^13^. The opening of deep sea between Antarctica, Australia and South America about 32 million years ago, and the subsequent onset of the Antarctic Circumpolar Current (ACC), initiated the formation of Antarctic icesheets and global cooling and drying^14^,^15^. Several episodes of relatively rapid cooling about 32, 14 and 5 Ma^16^,^17^ resulted in extinction of mesic-adapted lineages, such as gymnosperms^12^ and some mammals^18^. In Australia, the mid-Miocene marked an expansion of woodland, shrubland and grassland^16^ into regions that had been largely wet forest^19^,^20^. Strong aridification and expansion of the arid zone began about 15-14 Ma and intensified during the Pliocene (*ca.* 2–4 Ma)^16^,^21^,^22^ such that the semi-arid and arid zones (Eremaean) now cover about 70% of the Australian mainland^23^,^24^,^25^.

The origin of the savannah biome of the Australian monsoon tropics (AMT) is less well understood. Evidence from fossils and laterites indicates that seasonal climates were established in northern and central Australia by the time of the onset of the ACC^26^, however it appears that this was under temperate climate conditions and not monsoonal in the modern sense^27^. Savannah (open woodland with a C_4_ grassy understorey), the dominant ecosystem of the AMT, is generally thought to have originated globally within the last 10 million years, most likely between 8 and 5 Ma following the uplift of the Tibetan Plateau^28^,^29^, more frequent fire, and a rapid evolutionary and ecological expansion of C_4_ grasses^30^,^31^,^32^. These reconstructions lead to a prediction that the savannah biome of the AMT should have a more recent origin than the Eremaean (arid and semi-arid) shrublands that currently cover much of Australia, and we might expect that communities in the savannah biome assembled more recently than those of the shrublands.

Numerous studies have found lineage diversification coincident with global cooling and drying from the mid-Miocene in arid-distributed taxa (e.g.^21^,^33^,^34^,^35^,^36^,^37^). Crisp and Cook^27^ found that crown ages of sampled Australian plant lineages are congruent with the supposed ages of the biome in which the lineage occurs but, to date, there have been few tests of whether evolution of novel traits might have facilitated niche shifts into the newly expanding biomes.

Species of Triodiinae (*Triodia*, *Symplectrodia* and *Monodia*)^38^ are key components of the flora of the Australian Eremaean and savannah biomes, and exhibit remarkable morphological and physiological adaptations to extreme water deficit, high temperatures and oligotrophic soils, including cryptic stomata, leaf rolling and, in some species, well-developed sclerenchyma^39^,^40^. As such, Triodiinae is an excellent model for testing the directionality of niche shifts between arid and seasonal biomes and to assess if the evolution of novel traits might have allowed such niche shifts.

There are two major types of leaf anatomy in Triodiinae^10^,^41^, both of which roll to become needle-like: “amphistomatous”, in which stomata occur on both adaxial and abaxial surfaces, and “epistomatous”, in which stomata are restricted to the adaxial surface (Fig. 1). For grasses in general, it has been hypothesized that amphistomatous leaves that roll are an adaptation to environments with seasonal or diurnal fluctuations in water availability, whereas epistomatous leaves that roll might be advantageous in environments with sustained water stress by minimizing conductance^42^. Specimens of epistomatous Triodiinae usually also produce resin^43^ and have been called “soft” species because they are more palatable to livestock than the “hard” amphistomatous species^44^.

Here, we estimate the biogeographic history of Triodiinae to test whether it diversified in concert with major cooling periods in the mid-Miocene or early Pliocene. We use phylogenetic and ancestral-trait reconstruction methods to infer ancestral biomes and test whether the origins of novel leaf traits were coincident with biome shifts. If leaf traits were key innovations that allowed shifts between biomes, we would expect that the origin of novel leaf traits pre-date or coincide with biome shifts, and that those lineages with novel leaf traits increased in diversification rate coincident with, or shortly after, a biome shift. We also reassess the genus-level systematics of the group.

## Materials and Methods

### Taxon sampling and analysis

We sampled 145 individuals representing 66 of the 69 currently described species of Triodiinae^43^,^45^,^46^,^47^ (Supplementary data 1). Specimens were identified by MDC and JM and verified in the Australian National Herbarium specimen information register (ANHSIR, accessed 05/02/2013) or the relevant State Herbarium for non-data-based specimens. The cpDNA gene region *matK* and nuclear rDNA internal transcribed spacers (ITS) were amplified and sequenced for each specimen (see Supplementary data 2 for detailed methods).

### Phylogenetics

We combined our sequences of Triodiinae with those of outgroups so that the phylogeny could be rooted. Although the sister group of Triodiinae is unclear, the inclusion of Triodiinae within tribe Cynodonteae (subfamily Chloridoideae) is supported^7^,^48^. The outgroups, which included 46 species of Cynodonteae, 11 species of other Chloridoideae, and two more distant outgroups (*Centropodia glauca and Merxmuellera rangei,* Centropodieae), were obtained from GenBank (Supplementary data 2). The dataset was reduced to include only one representative of each haplotype (*matK*) or allele (ITS) for each species of Triodiinae.

To estimate phylogenetic relationships, we used maximum parsimony (MP), maximum likelihood (ML) and Bayesian with Markov chain Monte Carlo (MB) searches because each method has different underlying assumptions. We analysed each sequence dataset (*matK* and ITS) separately because each represents a different genome with different inheritance patterns (maternal and bi-parental respectively). We also performed a phylogenetic analysis using concatenated *matK* and ITS data. If results are congruent across different methods and genomic datasets, this increases our confidence in estimated relationships (see Supplementary data 2 for detailed methods).

### Molecular dating

We used BEAST^49^, which is a Bayesian method (MB) that simultaneously estimates model parameters, tree topology and divergence times, to estimate fossil-calibrated time trees of Triodiinae. BEAST can be used to compare alternative clock models using marginal likelihoods. We used a relaxed molecular-clock model of evolution because comparison of relative rate differences among lineages (Supplementary data 2) rejected a strict-clock model. Two analyses were run in BEAST using different relaxed clock models, Uncorrelated Lognormal (UCLN) and Random Local Clocks (RLC) and marginal likelihoods estimated using a path-sampling (PS) and a stepping-stone (SS) approach^50^,^51^ were compared using Bayes factors^52^,^53^.

### Diversification

Inferences of species diversification rates and trait evolution require phylogenetic trees with species-level sampling at the tips rather than including all individuals sampled (we had multiple samples for many species). For these analyses, posterior sets of BEAST trees were pruned with BayesTrees v1.1^54^ so that each monophyletic species was represented by only a single tip. For species that did not form a monophyletic group, each clade of samples identified to that species was represented by only one tip. The pruned ITS tree included 102 terminals, the *matK* tree included 95 terminals, and the concatenated pruned dataset included 81 terminals.

Lineage-through-time plots (LTTs) of estimated number of species were constructed for Triodiinae using 100 post-burn-in trees from each BEAST analysis (ITS, *matK,* concatenated) in Ape v3.0–11^55^. We also used the speciation-extinction model in BAMM^56^ and Bayes factors^52^,^53^ to test whether the diversification rate has been constant through time.

### Evolution of leaf anatomy

Free-hand sections, at least three per specimen, were made from the middle portion of leaves from fresh or herbarium-stored specimens and stained with Safranin. The position of vascular bundles was scored as central or near adaxial, and species were coded as either having stomatal grooves present on both leaf surfaces (amphistomatous or semi-amphistomatous) or only on the adaxial leaf surface (epistomatous). Several taxa that were identified as potential sister taxa based on the concatenated phylogenetic analyses, *Cleistogenes*, *Aeluropus*, *Dinebra*, *Vaseyochloa*, *Gouinia* and *Triplasis*, were scored for stomatal position using information available in the literature^57^.

Ancestral states for leaf traits were inferred using equal-weighted parsimony (Acctran and Deltran) in Mesquite v2.75^58^ separately for phylogenies derived using BEAST from each sequence dataset (ITS, *matK* and concatenated). To complement parsimony analysis and incorporate phylogenetic uncertainty, we also inferred ancestral states with the discrete-state continuous-time Markov chain (CTMC) model^59^ in BEAST with a symmetric substitution model and uninformative rate priors for state changes.

### Biogeography

Distributional data for each species were derived from the Atlas of Living Australia (http://www.ala.org.au, accessed 08/06/2013) (from 15,206 records of Triodiinae). Biome of occurrence (Eremaean, savannah and southern temperate)^13^,^22^,^60^ was scored for each species of Triodiinae using annual rainfall data sourced from the Australian government Bureau of Meteorology (http://www.bom.gov.au/jsp/ncc/climate_averages/rainfall/index.jsp, accessed 15/03/2014) to define the bounds of the Eremaean biome (< 500 mm annual average rainfall) and the tropic of Capricorn to delineate the southern temperate region from the savannah biome on the east coast of Australia. The tropic more or less coincides with the 80th percentile of summer (November-April) rainfall as a proportion of average annual rainfall, and was used to define the southern limit of the Australian Monsoon Tropics in a recent review^26^.

To infer the direction and timing of niche shifts, biome of occurrence was reconstructed using parsimony in Mesquite and model-based inference in BEAST, as described for the leaf traits. Because all potential outgroups are distributed outside Australia, they were excluded from ancestral state reconstruction of biomes.

### Leaf traits and biome shifts

We tested whether a shift in the position of vascular bundles is correlated with a change in the position of stomatal grooves, and whether changes in either leaf trait (positions of vascular bundles and stomatal grooves) are correlated with an inferred shift in biome of occurrence. These analyses required binary data, so we scored species distributions as either savannah biome or other (southern temperate and Eremaean regions), or as occurring in both categories. Only one species, *T. vella*, is restricted to the southern temperate region, six others (*T. longipalea*, *T. dielsii, T. danthonioides*, *T. compacta*, *T. irritans* and *T. scariosa*) occur in the southern temperate and Eremaean regions, and one species, *T. pungens*, is distributed across all three regions.

We tested correlations on the maximum clade credibility tree from the concatenated BEAST analysis using the Pagel94^61^ module in Mesquite v2.75^58^, with 1000 simulations each with 10 likelihood iterations. To incorporate error from tree estimation, we also tested correlations on 1000 trees from the posterior distribution from the BEAST analyses using the Discrete module in BayesTraits v2.0^62^, with all priors set to an exponential distribution with a mean of 10, and with 10^7^ reversible-jump MCMC iterations after burn-in^62^. The Discrete analysis compared independent (no correlation among shifts) and dependent (correlation among shifts) trait models using Bayes Factors^52^,^53^ calculated from harmonic means of the MCMC chains.

## Results

### Phylogenetics

**Figure 1:**
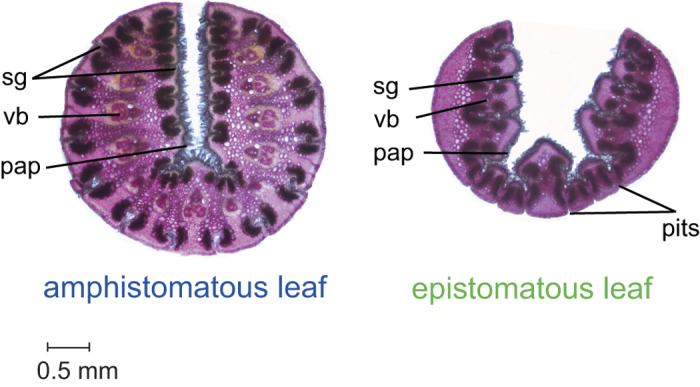
Amphistomatous and epistomatous leaves. Light microscopy images of cross-sections of rolled leaves of *Triodia* showing an amphistomatous leaf or “hard type” (*T. danthonioides*) and an epistomatous leaf or “soft type” (*T. bynoei*). The stomata are deeply sunken in longitudinal leaf grooves (sg) with dense interlocking papillae (pap). Note the pits near the midvein on the abaxial leaf surface of epistomatous species that are thought to allow leaf-rolling^41^. Vascular bundles (vb) are also shown.

The monophyly of Triodiinae was strongly supported by all analyses (MB PP = 1, ML BS ≥ 95, MP BS = 99) but no particular sister-group relationship was supported. *Triodia* was non-monophyletic in all analyses because both *Symplectrodia* and *Monodia* were nested within it (Fig. 1 and Supplementary data 3). All analyses of ITS and concatenated data recovered three major supported groups within Triodiinae (Clades I, II and III; Fig. 1 and Supplementary data 3). There was some supported conflict among phylogenies derived from ITS and *matK* for several taxa, leading us to exclude two species (*T. marginata* and *T. mitchellii*) and three other individuals (*T. triaristata* 4841685, *T. microstachya* 10237, *T. melvillei* 9785) from the concatenated dataset. Other minor discrepancies occurred only within clades and did not affect reconstructions (Supplementary data 3.1-3.4).

Triodiinae had significantly slower rates of molecular evolution than the other sampled chloridoid taxa (Supplementary data 2). The RLC clock model, which takes account of such variation, was decisively favoured (see Kass & Raftery^52^) for most analyses (2log_e_BF > 10) and strongly favoured (2log_e_BF = 5–10) for the others using both the Path-Sampling approach (concatenated dataset: 2log_e_BF = 85.1; ITS: 2log_e_BF = 17.4; *matK:* 2log_e_BF = 60.8) and the Stepping-Stone approach (concatenated: 2log_e_BF = 84.0; ITS: 2log_e_BF = 6.2; *matK*: 2log_e_BF = 59.8) (see Supplementary data 2 for a comparison of dating under the two models). All further analyses were therefore conducted on the RLC maximum clade credibility tree or RLC posterior set of trees. The crown age of Triodiinae was estimated to be 11.4–18.3 Ma (mean = 14.7 Ma) using the concatenated dataset and slightly older in analyses of the individual datasets (12.1–18.2 Ma, mean = 15.1 Ma, ITS; 14.2–21.9 Ma, mean = 18 Ma, *matK*), overlapping with the mid-Miocene (Fig. 1, Supplementary data 3). The stem age was estimated to be 17.9-23.5 Ma using the concatenated dataset, and dates estimated from the individual datasets (14–22.6 Ma, ITS; 17.5–22.5 Ma, *matK*) were mostly overlapping with this.

### Diversification of Triodiinae

**Figure 2:**
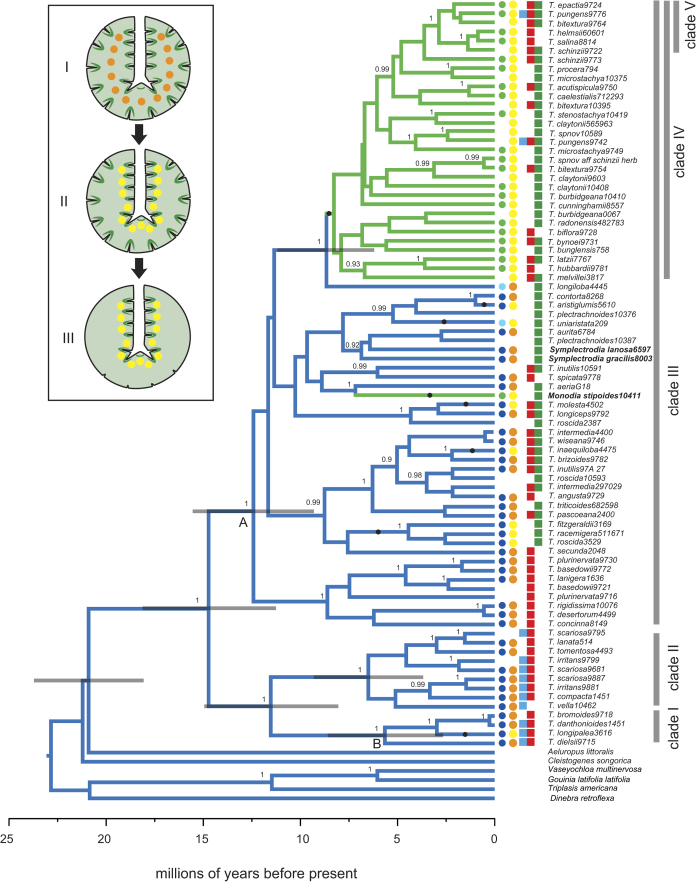
Timing and evolution of leaf traits of Triodiinae. Chronogram of concatenated ITS and *matK* datasets showing the node ages and relationships within Triodiinae inferred using BEAST with a Random Local Clocks (RLC) model. Only a single terminal is shown for species in which individuals form a supported monophyletic clade. Posterior probabilities ≥ 0.90 are shown on nodes. Node bars indicate 95% highest posterior density of age estimates. Branches are colour-coded by the character reconstruction of the position of leaf stomata: epistomatous (green) and amphistomatous (blue). Inferred transitions of central to near adaxial vascular bundles are shown as closed circles on branches. Earliest biome transitions from Eremaean to savannah (Node A) and Eremaean to temperate (Node B) are shown. Leaf traits and biome of each species are shown to the right of terminals. The first column shows position of leaf stomata: epistomatous (green), amphistomatous (blue) and semi-amphistomatous (pale blue). The second column shows vascular bundle position: central (orange) and near adaxial (yellow). The third to fifth columns shows occurrence in a biome: temperate (blue), Eremaean (red) and green (savannah). Inset illustrates the hypothesised transition of leaf traits in Triodiinae: (I) ancestral state of amphistomatous with central vascular bundles. (II) move of vascular bundles to near adaxial position. (III) loss of abaxial stomata (epistomatous) in two lineages.

The lineage-through-time plot (LTT) indicated an increase in diversification rate from the mid-Miocene, coincident with the period of cooling and drying after the mid-Miocene climatic optimum (Fig. 2 in McGowran *et al.*^16^), and then a progressively decreasing slope indicating a slowing rate from the Pliocene towards the present (Fig. 2). A two-step model with an increase in diversification rate at the crown of Triodiinae was strongly favoured (f = 0.83) over a model of constant diversification (f = 0.13, Bayes Factor = 24.4) by BAMM analyses, and were the only models represented in the 95% credible set of distinct shift configurations in BAMM (Supplementary data 3.9).

### Evolution of leaf traits

Most specimens were easily scored as amphistomatous or epistomatous (Supplementary data 1) except *T. uniaristata* and *T. longiloba* which both have stomatal grooves on both leaf surfaces (i.e., amphistomatous) but with none towards the abaxial leaf margins (semi-amphistomatous in Supplementary data 1). Two species, *T. pascoeana* and *T. aeria*, did not fit Lazarides′^43^ determination as “soft type” (epistomatous) and were coded here as amphistomatous (Supplementary data 1). All outgroups scored for stomatal position (*Cleistogenes*, *Aeluropus*, *Dinebra*, *Vaseyochloa*, *Gouinia* and *Triplasis*) were amphistomatous. In general, amphistomatous species had a larger proportion of sclerenchyma tissue than epistomatous species (Supplementary data 3.9).

Amphistomatous leaves were reconstructed as the ancestral state for Triodiinae in all analyses. All epistomatous species of *Triodia* form a clade (Clade IV) nested inside Clade III in analyses of ITS and the concatenated data (Fig. 2 and Supplementary data 3). The only other epistomatous species of Triodiinae, *Monodia stipoides*, is nested separately from Clade IV within Clade III in all analyses (Fig. 2 and Supplementary data 3). Thus, epistomatous leaves reconstruct as having at least two separate origins: once in the ancestor of Clade IV 9.5-5.4 Ma (Fig. 2) and another in the *Monodia stipoides* lineage. Trait reconstructions based on the *matK*-derived phylogeny estimated up to six origins of epistomatous leaf type, but this might be explained by low phylogenetic signal in the *matK* data because most nodes were not well supported. A central, rather than near adaxial, position of vascular bundles was inferred as the ancestral state in all analyses, with at least eight transitions from central to near adaxial (Fig. 2 and Supplementary data 3).

The positions of stomatal grooves and vascular bundles were strongly correlated (Pagel’s ML test *P* = 0.00; Bayesian MCMC test, 2log_e_BF = 18.6). All taxa with only adaxial stomatal grooves (epistomatous taxa: Clade IV and *Monodia*) have vascular bundles positioned near the adaxial leaf surface, whereas the position of vascular bundles varies among amphistomatous taxa: they are positioned either centrally or near the adaxial leaf surface (Tables 1 and 2). Apart from some members of Clade IV and *T. longipalea*, all taxa with vascular bundles near the adaxial surface occur in the savannah biome.

Two species (*T. uniaristata* and *T. longiloba*) are unusual in that they have abaxial stomatal grooves limited to near the mid-vein (semi-amphistomatous), with none near the abaxial leaf margin. One of these, *Triodia longiloba*, is sister to Clade IV in analyses of ITS and concatenated data and, although it has been considered a “soft” species (epistomatous), it has six pairs of abaxial stomatal grooves and centrally located vascular bundles with associated photosynthetic tissues (Supplementary data 1). The other anomalous species, *T. uniaristata*, is nested within a group of amphistomatous taxa from the Kakadu region of the [Fig f3]Northern Territory in all analyses (Fig. 4). It has only three pairs of abaxial stomatal grooves and has vascular bundles and associated photosynthetic tissues near the adaxial surface (Supplementary data 4).

### Leaf traits and biome shifts

The phylogeny of Triodiinae reveals geographic structuring (Fig. 4): Clade I is limited to southwest Australia and Clade II to southern Australia: together, they include the only species that extend from the Eremaean into the more mesic, temperate biome of southern Australia. Other clades are restricted to central Eremaean (Clade VII) or the far north of Australia, such as Clade IX in Kakadu, or more broadly distributed across both central Australia and the savannah biome (Clades IV and VIII).

The Eremaean biome was reconstructed as ancestral for Triodiinae, with multiple shifts or range expansions into the savannah biome from about 15.6-9.4 Ma, and into southern temperate biomes from about 8.9-2.9 Ma (Fig. 4). Ancestral biome reconstruction was robust to delineation of biome boundaries. Initial runs shifting the savannah/Eremaean boundaries by ± 100 mm annual average rainfall resulted in a few taxa being assigned to different biomes but no change in ancestral state reconstruction of the earliest transition from Eremaean to savannah (results not shown). Analyses using parsimony and Bayesian methods returned very similar reconstructions at nodes (Fig. 4), indicating that error arising from phylogenetic uncertainty or model choice did not affect reconstructions of ancestral biomes. The shifts into the savannah biome are strongly correlated with origins of epistomatous leaves (Pagel’s ML test *P* = 0.00; Bayesian MCMC test, 2log_e_BF = 25.2) and with the origins of near-adaxial vascular bundles (Pagel’s ML test *P* = 0.00; correlated using the Bayesian MCMC test, 2log_e_BF = 4.2). However, despite multiple inferred shifts or range expansions from the savannah back into the Eremaean biome within Clade IV (Fig. 4), there have been no reversals in leaf type (Tables 1 and 2), with all members of Clade IV retaining epistomatous leaves with adaxial vascular bundles.

## Discussion

The subtribe Triodiinae is monophyletic but *Triodia*, as currently recognized, is not. All analyses of both genome regions show support for the nesting of *Monodia* and *Symplectrodia* within *Triodia,* and we recommend synonymizing the three genera. Our findings also support Lazarides’^43^ synonymy of *Plectrachne* with *Triodia*: *T. schinzii* is nested inside Clade IV (Figs 2 and 4). Our phylogenetic evidence indicates that the synapomorphy for *Triodia*, multiple florets with more than one being fertile, is likely plesiomorphic for the subtribe and there have been shifts to different states in *Monodia* (single floret) and *Symplectrodia* (multiple florets with one fertile). We retain *Triodia* R.Br.^38^, giving it precedence over *Monodia* S.W.L.Jacobs, *Symplectrodia* Lazarides and *Plectrachne* Henrard.

### Diversification of Triodiinae

Our age estimates for Triodiinae preclude it being differentiated and present as a clade at the time Australia was still connected to Antarctica because even our oldest estimate for the stem age (24.5 Ma) is too young (Australia became completely isolated c. 33 Ma^63^). Triodiinae might have differentiated more recently from extinct Australian lineages or, more likely, arrived in Australia between 24.5 Ma and 14.0 Ma (95% HPD of stem) (Triodiinae is nested in a clade of Cynodonteae distributed in Asia, North America and Africa, with no other representatives native to Australia^7^). This is the period when narrowing distances between the Australian landmass and Southeast Asia appears to have facilitated dispersal of multiple plant groups^64^. The timing is consistent with the arrival of other species from the north that subsequently diversified in arid Australia, e.g., chenopods from Eurasia *ca.* 19.6-5 Ma^65^,^66^ (see^27^ for more examples) and in the savannah biome, e.g., *Livistona* palms from Asia *ca.* 16.8-5.9 Ma^67^.

**Figure 3 f3:**
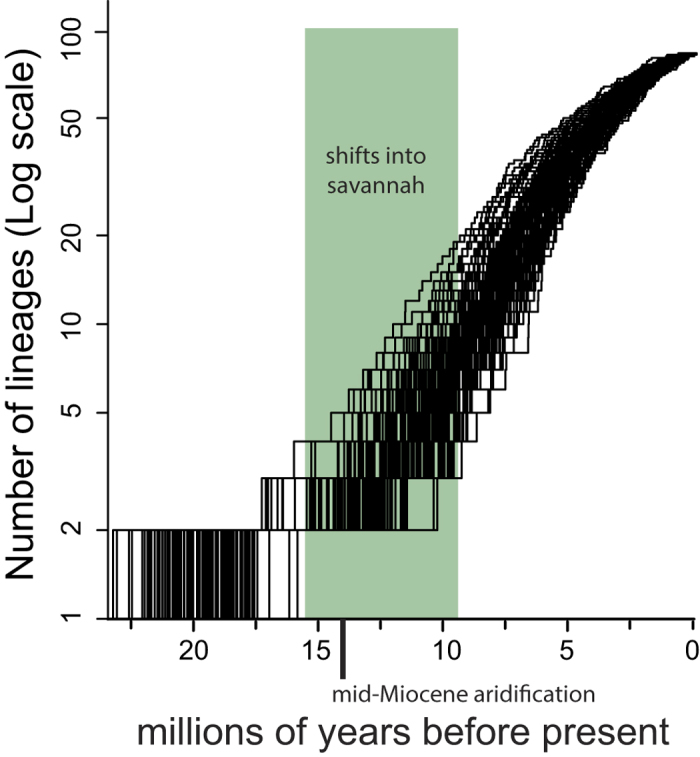
Diversification of Triodiinae. Lineages through time plots (LTT) showing diversification of Triodiinae over the past 25 million years (Ma) inferred from the BEAST concatenated (ITS and *matK*) analysis using a Random Local Clocks model (RLC). The timing of reconstructed shifts from the Eremaean biome into the savannah biome, and mid-Miocene aridification event, are indicated.

**Figure 4:**
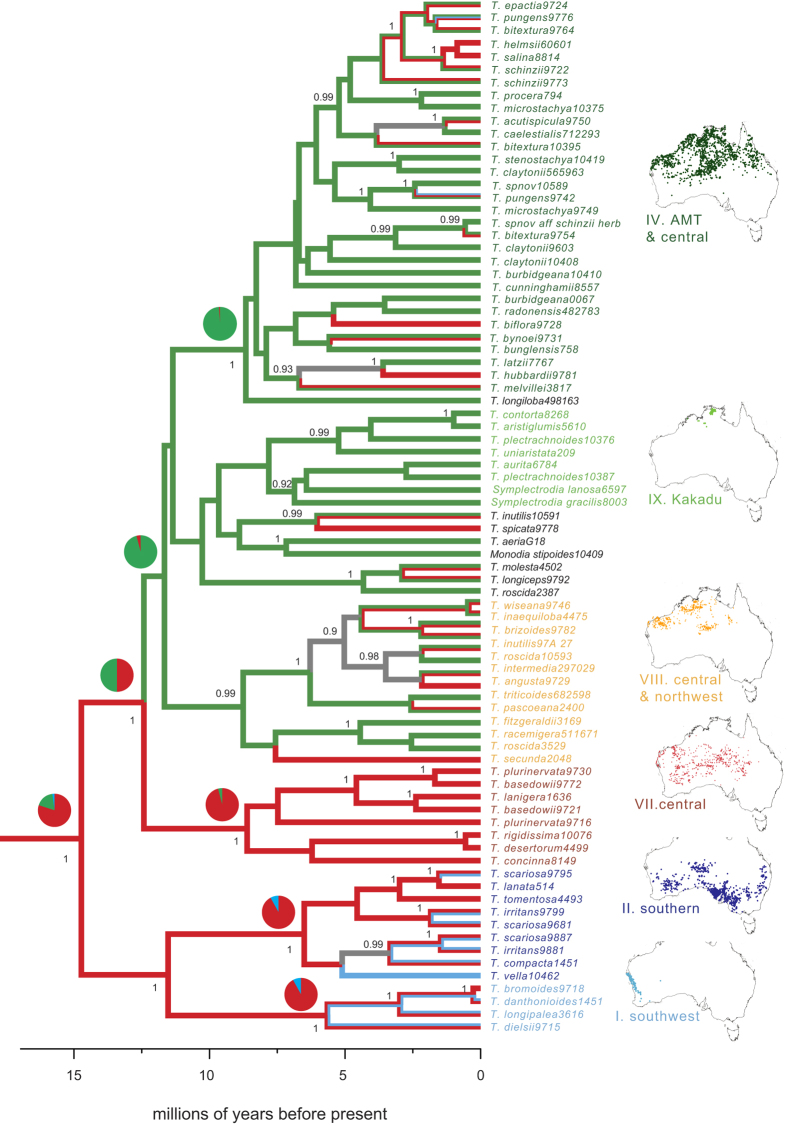
Biome shifts in Triodiinae. Chronogram of concatenated ITS and *matK* datasets showing biogeographic patterns of clades of Triodiinae inferred using BEAST with a Random Local Clocks (RLC) model. Only a single terminal is shown for species that form a supported monophyletic clade. Posterior probabilities ≥ 0.90 are shown on nodes. Branches are colour-coded by the character reconstruction of biome: temperate (blue), Eremaean (red) and savannah (green). Character states that are equivocal are coloured grey. Pies at nodes show posterior probabilities of biome reconstruction inferred using BEAST. Maps showing the distributions of taxa in clades were constructed using the Atlas of Living Australia (http://www.ala.org.au).

Triodiinae diversified rapidly coincident with the global mid-Miocene cooling and aridification (Fig. 3), when drier habitats expanded in Australia^16^. *Acacia*, another major lineage in the Australian Eremaean biome also diversified rapidly during this time^68^. Our analysis found support for a significant shift in rate of diversification contemporaneous with the mid-Miocene drying event, supporting the hypothesis that diversification was coincident with the rapidly intensifying aridification of Australia. The LTT plot shows a relatively even rate of diversification after this time then slowing towards the present, which might indicate progressive niche saturation, recent extinction or incomplete sampling of tip taxa^69^.

### Key innovations or subsequent adaptive change

Multiple reconstruction methods identified the Eremaean biome as ancestral for Triodiinae, rather than the southern temperate region biome, at odds with an expectation that more mesic regions are ancestral for Australian plants (see^13^). However, this expectation should apply only to those lineages that were present in Australia at the time it became isolated from the rest of Gondwana, as this is the time that the landmass was largely mesic^13^. If Triodiinae dispersed into Australia during the Miocene, as estimated here, it might already have been already adapted to warm arid climates. No studies to date^7^,^48^ have identified clear sister relationships for Triodiinae. Therefore, is not yet possible to determine from where it might have dispersed.

We reconstructed multiple shifts from the Eremaean biome into the savannah biome, coincident with the expansion of savannah biomes globally in the past 10 Ma^28^,^29^. If these shifts led to adaptive radiations in the newly colonised biome, we would expect there to be a signal of increased diversification in the lineage making that shift. However, the BAMM analyses showed no indication of an increase in diversification in Clade III, the lineage making the shift, with the only supported rate-shift being at the crown of Triodiinae, i.e., the mid-Miocene rate-shift coincident with aridification.

For plants in general, shifts between savannah and arid ecosystems have been estimated to be relatively rare^70^, indicating that many species might recognise strong ecological differences between these habitats. In contrast, we estimated many shifts between the two biomes in Triodiinae (66% of all shifts) indicating that such shifts have been relatively easy in this group. Although the Eremaean and savannah biomes both have drought, the savannah biome experiences a relatively reliable and predictable alternation between drought and high precipitation compared with the Eremaean zone^60^. The correlation between leaf traits and biome of occurrence indicates that the relevant selective force might have been changes in the predictability of rainfall (seasonal rainfall versus infrequent).

Both amphistomatous and epistomatous leaves can roll and unroll according to conditions although, generally, the more heavily sclerified the leaf tissue, the less open the blade when fresh, and some amphistomatous species appear permanently rolled^71^. Epistomatous taxa, with less-sclerified leaf tissue, might have a greater capacity for unrolling and rolling. When water resources are not limiting, for example during the summer-wet period, the epistomatous leaf type is unrolled (P. Davidson, pers. comm.) and the stomata exposed to allow gas exchange. In this situation, the close proximity of the adaxial vascular bundles to the stomata might enable prompt export of photosynthates to the phloem, away from herbivore attack, and towards increased root growth or storage.

In contrast, the amphistomatous leaf anatomy appears to represent a more generalist physiological strategy and, indeed, we reconstruct this form as the ancestral type for Triodiinae. The amount of intrusive sclerenchyma in the amphistomatous leaf would reduce incoming light as well as limit the exchange of solutes between bundle and chlorenchyma (D. Gaff, pers. com.; see^72^). However, the presence of abaxial stomata should facilitate gas exchange while minimising water loss by retaining a rolled leaf, enabling them to successfully withstand arid conditions with highly infrequent rainfall. Nevertheless, the taxa in Clade IV that appear to have shifted from the savannah biome back into the Eremaean zone also survive well in that habitat with an epistomatous leaf form. Fine-scale modeling of species distributions, or local-scale observations, could assess whether these taxa occupy microhabitats with greater water availability than those the amphistomatous species occupy. For example, inland populations of *T. schinzii* (epistomatous) are associated with sand dunes, which have higher water availability than nearby inter-dunes in which occurs an amphistomatous species, *T. basedowii*^73^.

The order of biome and leaf-shape shifts is apparent from trait mapping on the phylogenies (also see Tables 1 and 2). The ancestral Triodiinae was likely amphistomatous, with central vascular bundles and lived in an Eremaean environment. A shift from the Eremaean biome into the savannah biome predates a move of vascular bundles from a central position to being near adaxial. An independent move of vascular bundles from a central to near adaxial position has occurred in *T. longipalea*, a lineage that occurs in the Eremaean biome and the temperate region of SW WA rather than the savannah biome. Following the move of vascular bundles towards the inner surface, two lineages in the savannah biome (Clade IV and *Monodia*) have then lost stomata from the outer (abaxial) surface.

Although correlated with lineages that have moved into a new biome (savannah) with different rainfall regimes from that of their ancestors (Eremaean biome), the two leaf traits were not key innovations that allowed the shifts—the changes in leaf morphology followed the biome shift rather than enabled it. There was no change in the diversification rate in lineages evolving the new traits (no rate shifts detected by BAMM analyses), further indicating that these traits did not enable an adaptive radiation. However, the multiple independent moves of vascular bundles towards the inner surface in lineages in the savannah biome are suggestive of it being adaptive for taxa in that biome.

## Conclusions

Triodiinae has radiated in concert with a drying climate in Australia, with species then shifting into the savannah with expansion of that biome. Evolution of leaf anatomy is correlated with these niche shifts, but appears to have occurred after the shifts rather than being key innovations that allowed expansion into the savannah environment. This does not preclude the possibility that other key innovations might have facilitated the shifts. Adaptation to drought and an ability to respond quickly to rainfall might have provided a pre-adaption for Triodiinae to move into the highly seasonal savannah biome as it expanded over the past 10 Myr. Physiological evaluation of epistomatous and amphistomatous species should assist in understanding how leaf properties are linked to plant function and evolutionary relationships of this group.

*Triodia* as currently circumscribed is not monophyletic, with *Monodia* and *Symplectrodia* nested within it. All three genera should be synonymised under *Triodia*.

## Additional Information

**Accession codes:** Sequences collected in this study were deposited in GenBank under the accession numbers KT199427 - KT199706.

**How to cite this article**: Toon, A. *et al.* Key innovation or adaptive change? A test of leaf traits using Triodiinae in Australia. *Sci. Rep.* **5**, 12398; doi: 10.1038/srep12398 (2015).

## Supplementary Material

Supplementary Data 1-4

## Figures and Tables

**Table 1:** The number of trait shifts of leaf vascular bundle position (central or near adaxial) in Triodiinae that are coincident with the ancestral state reconstruction of stomatal groove position (epistomatous or amphistomatous) and biome (savannah, Eremaean, temperate).

trait shift	stomatal groove reconstruction		biome reconstruction					
vascular bundles	epistomatous	amphistomatous	savannah	savannah/Eremaean	Eremaean	Eremaean/temperate	temperate	total
central→adaxial	2	6	5	2	0	1	0	8
adaxial→central	0	0	0	0	0	0	0	0

**Table 2:** The number of trait shifts of leaf stomatal groove position (epistomatous or amphistomatous) in Triodiinae that are coincident with the ancestral state reconstruction of vascular bundle position (central or near adaxial) and biome (savannah, Eremaean, temperate).

trait shift	vascular bundles reconstruction		biome reconstruction					
stomatal grooves	central	near adaxial	savannah	savannah/Eremaean	Eremaean	Eremaean/temperate	temperate	total
amphistomatous→epistomatous	0	2	2	0	0	0	0	2
epistomatous→amphistomatous	0	0	0	0	0	0	0	0
