# Supplementary material for: Key innovation or adaptive change? A test of leaf traits using Triodiinae in Australia
Source: Sci Rep. 2015 Jul 28;5:12398. doi: 10.1038/srep12398 (PMC4648476; doi:10.1038/srep12398)
Supplement: Supplementary Data 1-4 [file srep12398-s1.pdf]

Key innovation or adaptive change? A test of leaf traits using Triodiinae in Australia

Toon, A.[1*], Crisp, M.D.[2], Gamage, H.[3], Mant, J.[1,2], Morris, D.C.[2], Schmidt, S.[3], Cook, L.G.[1]

[1]School of Biological Sciences, The University of Queensland, Brisbane QLD 4072, Australia.

[2]Research School of Biology. The Australian National University, Research School of Biology, Canberra ACT 2601, Australia.

[3]School of Agriculture and Food Sciences, The University of Queensland, Brisbane QLD 4072, Australia.

Supplementary Data 1: Species of Triodiinae used in this study organised by clade (Figure 2) and, for those examined anatomically, the arrangement of stomatal grooves and vascular bundle arrangement is indicated. Specimen determination was obtained from the Australian National Herbarium specimen information register (ANHSIR) or the relevant State Herbarium for non-data-based specimens. The classification to informal infragenus (species group) of Lazarides (1997) is indicated for each genus.

Species	Herbarium ID	Species group	Stomatal grooves	Vascular bundles
**Clade I**				
T. bromoides	Crisp 9718	Danthoniodies	amphistomatous	Central
T. danthonioides	Donaldson 1451	Danthoniodies	amphistomatous	Central
T. danthonioides	BRI AQ0681932	Danthoniodies		
T. dielsii	Crisp 9715	Danthoniodies	amphistomatous	Central
T. dielsii	Crisp 6281	Danthoniodies		
T. longipalea	Lepschi 3616	Danthoniodies	amphistomatous	Near adaxial
**Clade II**				
T. scariosa+	Crisp 9887	Scariosa	amphistomatous	Central
T. scariosa+	Crisp 9804	Scariosa		
T. scariosa	Crisp 9681	Scariosa	amphistomatous	Central
T. scariosa	Crisp 9795	Scariosa		
T. compacta	Fed 1451	Scariosa	amphistomatous	Central
T. compacta	Fed 1455	Scariosa		
T. irritans	Crisp 9881	Scariosa	amphistomatous	Central
T. irritans	Crisp 9799	Scariosa		
T. lanata	Hadlow 514	Scariosa	amphistomatous	Central
T. lanata	NPWS 1601	Scariosa		
T. lanata	BRI AQ0426549	Scariosa		
T. tomentosa	Lepschi 4493	Scariosa	amphistomatous	Central
T. vella	Crisp 10462	Scariosa	amphistomatous	Central
**Clade III**				
T. angusta	Crisp 9729	Angusta	amphistomatous	Central
T. fitzgeraldii	Lazarides 3169	Angusta	amphistomatous	Near adaxial
T. racemigera	Craig 00511671	Angusta	amphistomatous	Near adaxial
T. racemigera	Crisp 10398	Angusta		

T. racemigera	Crisp 10401	Angusta		
T. racemigera	Crisp 10537	Angusta		
T. racemigera	PERTH 6064310	Angusta		
T. secunda	Davidson 2048	Angusta	amphistomatous	Central
T. triticoides	BRI AQ0682598	Angusta	amphistomatous	Central
T. basedowii	Crisp 9772	Basedowii	amphistomatous	Central
T. basedowii	Crisp 9721	Basedowii		
T. basedowii	BRI AQ0451359	Basedowii		
T. brizoides	Crisp 9782	Basedowii	amphistomatous	Central
T. intermedia	Rice 4400	Basedowii	amphistomatous	Central
T. intermedia	BRI AQ0297029	Basedowii		
T. inutilis	Wallis 97A/27	Basedowii	amphistomatous	Near adaxial
T. inutilis	Crisp 10591	Basedowii		
T. lanigera	Mitchell PRP 1636	Basedowii	amphistomatous	Central
T. molesta	Rice 4502	Basedowii	amphistomatous	Near adaxial
T. molesta	Crisp 10485	Basedowii		
T. roscida	Lazarides 2387	Basedowii		
T. roscida	Dunlop 3529	Basedowii	amphistomatous	Near adaxial
T. roscida	Crisp 10593	Basedowii		
T. wiseana	Crisp 9746	Basedowii	amphistomatous	Central
T. desertorum	Lepschi 4499	Danthonioides	amphistomatous	Central
T. rigidissima	Crisp 10076	Danthonioides	amphistomatous	Central
T. rigidissima	Crisp 10724	Danthonioides		
T. aristiglumis	Cowie 5610	Plectrachnoides	amphistomatous	Near adaxial
T. aurita	Carolin 6784	Plectrachnoides	amphistomatous	Central
T. contorta	Cowie 8268	Plectrachnoides	amphistomatous	Central
T. contorta	Cowie 8674	Plectrachnoides		
T. contorta	BRI AQ0580722	Plectrachnoides		
T. plectrachnoides	Crisp 10376	Plectrachnoides	amphistomatous	Central
T. plectrachnoides	Crisp 10387	Plectrachnoides		
T. plectrachnoides	BRI AQ0513279	Plectrachnoides		
T. uniaristata	Hinz R 209	Plectrachnoides	semi-amphistomatous*	Near adaxial
T. uniaristata	Craven 8128	Plectrachnoides		
T. uniaristata	Dunlop 6329	Plectrachnoides		
T. pascoeana	Mollemans 2400	Procera	amphistomatous	Central
T. aeria	Marshall G 18	Schinzii	amphistomatous	Central
T. aeria	Crisp 10460	Schinzii		
T. concinna	George 8149	Spicata	amphistomatous	Central
T. concinna	PRP1218	Spicata		
T. inaequiloba	Rice 4475	Spicata	amphistomatous	Central
T. integra	Beauglehole 426552	Spicata	amphistomatous	Central
T. longiceps	Crisp 9792	Spicata	amphistomatous	Central
T. longiceps	Crisp 9725	Spicata		
T. longiceps	Crisp 9768	Spicata		
T. plurinervata	Crisp 9730	Spicata	amphistomatous	Central
T. plurinervata	Crisp 9716	Spicata		

T. spicata	Crisp 9778	Spicata	amphistomatous	Central
T. spicata	BRI AQ426573	Spicata		
Monodia stipoides	Crisp 10409	Monodia	epistomatous	Near adaxial
Monodia stipoides	Crisp 10411	Monodia		
Symplectrodia lanata	Dunlop 6597	Symplectrodia	amphistomatous	Central
S. gracilis	Lazarides 8003	Symplectrodia	amphistomatous	Central
**Clade III-IV**				
T. biflora	Crisp 9728	Procera	epistomatous	Near adaxial
T. burbidgeana	TH0067S	Procera		
T. burbidgeana	Crisp 10410	Procera	epistomatous	Near adaxial
T. cunninghamii	Lazarides 8557	Procera	epistomatous	Near adaxial
T. latzii	Albrechat 7767	Procera	epistomatous	Near adaxial
T. microstachya	Crisp 9749	Procera	epistomatous	Near adaxial
T. microstachya	Crisp 9738	Procera		
T. microstachya	Crisp 9761	Procera		
T. microstachya	Crisp 10237	Procera		
T. microstachya	Crisp 10375	Procera		
T. procera	Waddy 794	Procera	epistomatous	Near adaxial
T. radonensis	Latz 482783	Procera	epistomatous	Near adaxial
T. stenostachya	Crisp 10419	Procera	epistomatous	Near adaxial
T. stenostachya	Crisp 9604	Procera		
T. epactia	Crisp 9724	Pungens	epistomatous	Near adaxial
T. epactia	Crisp 9723	Pungens		
T. epactia	Crisp 9733	Pungens		
T. hubbardii	Crisp 9781	Pungens	epistomatous	Near adaxial
T. longiloba	CANB 498163	Pungens	semi-amphistomatous*	Central
T. longiloba	Crisp 10415	Pungens		
T. marginata	Canning 6073	Pungens	epistomatous	Near adaxial
T. mitchellii	Crisp 10208	Pungens	epistomatous	Near adaxial
T. mitchellii	Crisp 9571	Pungens		
T. mitchellii	Crisp 9605	Pungens		
T. mitchellii	Crisp 9609	Pungens		
T. mitchellii	Crisp 10474	Pungens		
T. mitchellii	QH 572642	Pungens		
T. pungens	Crisp 9776	Pungens	epistomatous	Near adaxial
T. pungens	Crisp 9742	Pungens		
T. pungens	Crisp 9765	Pungens		
T. pungens	Crisp 10195	Pungens		
T. pungens	Crisp 10305	Pungens		
T. pungens	Crisp 10309	Pungens		
T. pungens	Crisp 9583	Pungens		
T. pungens	Crisp 9585	Pungens		
T. acutispicula	Crisp 9750	Schinzii	epistomatous	Near adaxial
T. acutispicula	BRI AQ0469248	Schinzii		
T. bitextura	Crisp 9754	Schinzii	epistomatous	Near adaxial
T. bitextura	Crisp 9748	Schinzii		
T. bitextura	Crisp 9756	Schinzii		
T. bitextura	Crisp 9764	Schinzii		

T. bitextura	Crisp 9767	Schinzii		
T. bitextura	Crisp 10395	Schinzii		
T. bitextura	Crisp 10397	Schinzii		
T. bitextura	Crisp 10400	Schinzii		
T. bitextura	BRI AQ0586960	Schinzii		
T. bunglensis	Menkhorse 758	Schinzii	epistomatous	Near adaxial
T. bunglensis	Crisp 10592	Schinzii		
T. bunglensis	Crisp 10595	Schinzii		
T. bynoei	Crisp 9731	Schinzii	epistomatous	Near adaxial
T. bynoei	BRI AQ0599864	Schinzii		
T. caelestialis	CANB 712293	Schinzii	epistomatous	Near adaxial
T. caelestialis	Crisp 10458	Schinzii		
T. claytonii	Crisp 10408	Schinzii	epistomatous	Near adaxial
T. claytonii	Crisp 10413	Schinzii		
T. claytonii	Crisp 9603	Schinzii		
T. claytonii	BRI AQ0565963	Schinzii		
T. helmsii	Beauglehole 60601	Schinzii	epistomatous	Near adaxial
T. melvillei	Crisp 9785	Schinzii	epistomatous	Near adaxial
T. melvillei	van Leeuwen 3817	Schinzii		
T. prona	Lazarides 6715	Schinzii	epistomatous	Near adaxial
T. salina	Albrecht 8814	Schinzii	epistomatous	Near adaxial
T. schinzii	Crisp 9773	Schinzii	epistomatous	Near adaxial
T. schinzii	Crisp 9722	Schinzii		
T. sp. aff. claytonii	Crisp 10418	Schinzii	epistomatous	Near adaxial
T. sp. nov. (aff.T. schinzii)	Fresh sample	Schinzii	epistomatous	Near adaxial
T. triaristata	CANB 484168	Schinzii	epistomatous	Near adaxial

+ previously *T. bunicola* (Hurry et al. 2012)

* semi-amphistomatous have abaxial stomatal grooves limited to near the mid-vein

Supplementary Data 2: Methods and Results

DNA Amplification and sequencing

DNA was extracted from leaf samples stored in silica gel or on herbarium sheets by homogenising with a Qiagen TissueLyser, then using a Qiagen DNeasy Plant Minikit following the manufacturer's protocol. The cpDNA gene region *matK* was sequenced using the Hilu[1] primers S5-1F (5'-ACCCTGTTCTGACCATATTG) and 9R (5'-TACGAGCTAAAGTTCTAGC), with internal primers (W 5'-TACCCTATCCTATCCAT and 1210R 5'-GTAGTTGAGAAAGAATCGC) used to sequence some degraded samples. The nuclear rDNA internal transcribed spacers (ITS) were amplified using primers P1L (5'-CTGTAGGTGAACCTGCGGAAGGATC) and P2R (5'-CTTTTCCTCCGCTTATTGATA)[2]. Standard PCR was carried out as described in Cook & Crisp[3] with 50°C and 55°C annealing temperatures respectively for *matK* and ITS. ITS sequences with multiple polymorphic sites were cloned using PGEM-T vector (Promega) and 6-8 colonies per specimen were sequenced.

Sequences were edited using SEQUENCHER 4.7 (Gene Codes Corporation, MI, USA) and checked for base composition bias using PAUP* v4.0b10[4]. They were aligned using MAFFT v6[5] (http://mafft.cbrc.jp/alignment/software/) using the simple FFT-NS-I algorithm for *matK* and Q-INS-I, which considers secondary structure, for ITS.

Phylogenetics

All analyses (individual and concatenated) were partitioned by DNA region (*matK*, ITS) to incorporate individual evolutionary models. Maximum likelihood analyses were conducted in RAxML v7.2.7 [6,7] with a GTRGAMMA model for each data partition and the rapid search option. Node support was estimated in final runs with 1000 bootstrap pseudoreplicates. Bayesian searches were run in MrBayes 3.2[8] with a GTR+I+G model for each data partition selected using MrModelTest[9] and with two runs and four chains sampling every 1000 of 4 x 10^7 generations. Trees were compared across runs for convergence using the comparison of split frequencies and symmetric tree-difference scores in AWTY[10,11]. Burn-in comprised 25-50% of

samples depending on results from AWTY and convergence of harmonic means, and combined runs were searched for the maximum clade credibility tree (maximum product of the posterior clades) in TREEANNOTATOR[12].

Within Triodiinae, the ITS (591 base pairs) and *matK* (1253 base pairs) alignments had 188 and 91 variable sites respectively, of which 133 and 43 respectively were parsimony informative (sequence accession's KT199427-KT199706). No base composition bias was detected within the Chloridoideae for either DNA region (P = 1.0).

Relative rates of molecular evolution

Conserved substitutional rate heterogeneity among lineages has the potential to affect age estimates under some clock models[13,14]. We compared the rate of molecular evolution in Triodiinae to other chloridoids using Fisher's exact and Mann-Whitney U tests implemented in GraphPad Prism 5.03 (GraphPad Software 2009). Uncorrected genetic distances (p-distance) were calculated in PAUP* between Triodiinae or chloridoids, and each of the 10 outgroups (see Table 1 below), selected randomly using the random number generator in Microsoft Excel 14.4.1 (2010), and average rates of divergence between each group were tested for significant differences.

Triodiinae had significantly slower rates of molecular evolution than the other sampled chloridoid taxa (ITS: t-test $P < 0.01$, Mann-Whitney U $P < 0.01$; *matK*: t-test $P < 0.01$, Mann-Whitney U $P < 0.01$).

Molecular Dating

Two analyses were run in BEAST using different relaxed clock models, Uncorrelated Lognormal (UCLN) and Random Local Clocks (RLC). The RLC model accounts for sudden shifts in clock rates and assumes autocorrelation of rates between shifts[15], and is a more appropriate model to use when clock rate is correlated within lineages but differs among lineages[13,14]. UCLN assumes no rate correlation between adjacent branches and is the preferred model in many circumstances[16]. We statistically compared the fit of the RLC and UCLN models using log Bayes

factors[17,18] calculated from marginal likelihoods estimated using a path-sampling (PS) and, alternatively, a stepping-stone (SS) approach in BEAST[19,20].

To calibrate the molecular clocks to absolute time, we used a secondary calibration for the most recent common ancestor (crown node) of Chloridoideae, which was 32.0 (±3.8) Ma, consistent with recent fossil-calibrated dating of Poaceae (28.2-35.8 Ma[21]; 28.9-33.2 Ma[22]). A published phytolith fossil[23] has been used to calibrate the age of the Chloridoideae at *ca* 20 Ma older than the secondary calibration above but it was not used here because it conflicts with other fossil-calibrated divergences[21,24] in Poaceae and consequently fails the cross-validation test[25].

We excluded several specimens (*T. triaristata* 4841685, *T. microstachya* 10237, *T. melvillei* 9785, *T. marginata* and *T. mitchelii*) from the concatenated ITS-*matK* dataset because their relationships differed significantly in ITS and *matK* analyses. Two runs were performed with each clock model using a speciation model of birth-death with incomplete sampling[26], a random starting tree and a GTR+I+G model (selected using MrModelTest[9]) for each partition (*matK*, ITS). BEAST analyses were run for 4 x 10^8 generations, sampling every 10,000 generations. To check efficiency of the Bayesian searches, stationarity and mixing of parameter values were assessed in TRACER v1.5. The maximum clade credibility (optimal) tree from each search was extracted using TREEANNOTATOR[12] from the posterior set of 20,000 trees, having discarded the "burn-in" comprising results preceding stationarity of the MCMC chain. PS and SS sampling of marginal likelihoods consisted of a chain length of 500,000 steps with 100 path steps and a β (0.3, 1.0) path scheme.

Comparison of RLC and UCLN model

The RLC clock model and the UCLN model estimated very different ages for the crown age of Triodiinae. The UCLN model returned crown-age estimates of 4.5-8.3 Ma (mean = 6.3 Ma) from the concatenated dataset and similar from individual datasets (4.2-8.7 Ma, mean = 6.5 Ma, ITS; 4.5-11.6 Ma, mean = 7.6 Ma, *matK*), bridging the Miocene-Pliocene boundary. In contrast, the RLC model gave estimates of 11.4-18.3 Ma (mean = 14.7 Ma) for the concatenated dataset and slightly older for

the individual datasets (12.1-18.2 Ma, mean = 15.1 Ma, ITS; 14.2-21.9 Ma, mean = 18 Ma, *matK*), overlapping with the mid-Miocene (Supplementary data 3). Stem ages were less affected by model choice than crown ages, with estimates from the RLC model (17.9-23.5 Ma, concatenated; 14-22.6 Ma, ITS; 17.5-22.5 Ma, *matK*) mostly overlapping those from the UCLN model (10.3-21.9 Ma, concatenated; 9.8-22.2 Ma, ITS; 12.4-21.3 Ma, *matK*) (Supplementary data 3). The RLC model was decisively favoured for most analyses (2log$_e$BF > 10), and strongly favoured (2log$_e$BF = 5-10) for the others see [17], using both the Path-Sampling approach (concatenated dataset: 2log$_e$BF = 85.1; ITS: 2log$_e$BF = 17.4; *matK:* 2log$_e$BF = 60.8) and the Stepping-Stone approach (concatenated: 2log$_e$BF = 84.0; ITS: 2log$_e$BF = 6.2; *matK*: 2log$_e$BF = 59.8).

Table 1: Poaceae dataset used as outgroups for molecular dating of Triodiinae. Genbank accession numbers are given for ITS and *matK.* Randomly selected outgroups used in the relative rates analysis of Triodiinae and other chloridoids are indicated with $_{RR}$.

outgroups	ITS	*matK*
Acrachne racemosa	AY574635	JN681616
Aegopogon cenchroides	EF153020	AF312324
Aeluropus littoralis	GU359262	AF144597
Amphipogon caricinus $_{RR}$	AF019849	AF312794
Astrebla lappacea	AF019838	AF144589
Austrodanthonia tenuior $_{RR}$	EU401279	HE574412
Blepharidachne kingie	EF153023	JN681618
Bouteloua curtipendula	EF060127	AF144578
Centropodia glauca $_{RR}$	AF019861	AF164410
Chionochloa rubra $_{RR}$	EU401294	EU400722
Chloris elata	EF153031	HE573964
Chloris truncata	AF019840	AF312330
Cleistogenes songorica	FJ548983	AF164428
Cottea pappophoroides	EF153033	AF312359

Cynodon dactylon	AF019839	AF144584
Cynodon transvaalensis	AF058808	AF312331
Dactyloctenium aegyptium	GU359251	HE573967
Dasyochloa pulchella	GU359330	JN681619
Dinebra retroflexa	AY576670	AF144594
Distichlis spicata	EF153040	HE573968
Eleusine coracana	AY515200	HQ180864
Eleusine tristachya	AY515223	FN908053
Enneapogon scoparius	DQ655843	AF312361
Enteropogon macrostachyus	GU359340	JN681630
Eragrostis echinochloidea	DQ655818	AF144605
Eustachys distichophylla	EF153051	AF144586
Gouinia latifolia	EF153053	JN681633
Gynerium sagittatum $_{RR}$	AF019858	HE586080
Harpochloa falx	DQ655800	JN681620
Hilaria cenchroides	GU359143	JN205313
Joinvillea plicata $_{RR}$	AF019784	DQ257535
Karroochloa curva $_{RR}$	DQ218200	EU400735
Leptochloa dubia	GU359145	AF312344
Lepturus repens	GU359150	AF144598
Lintonia nutans	GU359151	AF312337
Melanocenchris abyssinica	EF153063	AF312326
Microchloa caffra	GU359155	AF164425
Monanthochloe littoralis	EF196899	AF312349
Munroa squarrosa	EF153069	JN681623
Neobouteloua lophostachya	GU359123	JN681639
Neostapfia colusana	EF153071	AF312351
Orcuttia californica	EF153072	AF144599
Oryza sativa $_{RR}$	DQ355273	AF148650
Pappophorum vaginatum	EF153073	JN681624
Perotis patens	DQ655812	FR821323
Perotis rara	GU359134	AF144590
Reederochloa eludens	EF153077	AF312350

Schedonnardus paniculatus	GU359201	AF312335
Schmidtia pappophoroides	DQ655844	AF312362
Scleropogon brevifolius	GU359203	JN681625
Spartina pectinata	EF153082	AF312353
Sporobolus indicus	EF153083	AF144601
Streptochaeta sodiroana RR	AF019785	HE573921
Swallenia alexandrae	GU359217	JN681626
Tragus berteronianus	DQ655808	AF144591
Trichloris crinita	EF153088	AF144588
Tridens flavus	EF153090	AF312346
Triplasis americana	EF153093	JN681642
Tuctoria greenei	GU359190	AF312352
Uniola paniculata	EF153096	AF144607
Vaseyochloa multinervosa	GU359193	AF312348
Willkommia texana	EF153098	AF312348
Zeugites pittieri RR	AM404342	AF144576
Zoysia japonica	HQ600481	HE573979

References

1 Hilu, K. W. & Alice, L. A. Evolutionary implications of matK indels in Poaceae. *Am. J. Bot.* **86**, 1735–1741, (1999).

2 Crisp, M. D., Gilmore, S. R. & Weston, P. H. Phylogenetic relationships of two anomalous species of *Pultenaea* (Fabaceae: Mirbelieae), and description of a new genus. *Taxon* **48**, 701-714, (1999).

3 Crisp, M. D. & Cook, L. G. Explosive radiation or cryptic mass extinction? Interpreting signatures in molecular phylogenies. *Evolution* **63**, 2257-2265, (2009).

4 Swofford, D. L. PAUP*: phylogenetic analysis using parsimony (* and other methods). v.4.0b10. Sinauer, Sunderland, MA., (2002).

5 Katoh, K. & Toh, H. Recent developments in the MAFFT multiple sequence alignment program. *Brief. Bioinform.* **9**, 286-298, (2008).

6 Stamatakis, A. RAxML-VI-HPC: maximum likelihood-based phylogenetic analyses with thousands of taxa and mixed models. *Bioinform.* **22**, 2688-2690, (2006).

7 Stamatakis, A., Hoover, P. & Rougemont, J. A rapid bootstrap algorithm for the RAxML web servers. *Syst. Biol.* **57**, 758-771, (2008).

8 Ronquist, F. *et al.* MrBayes 3.2: efficient Bayesian phylogenetic inference and model choice across a large model space. *Syst. Biol.* **61**, 539-542, (2012).

9 Nylander, J. A. A. MrModeltest v2. Program distributed by the author. Evolutionary Biology Centre, Uppsala University. (2004).

10 Wilgenbusch, J. C., Warren, D. L. & Swofford, D. L. AWTY: a system for graphical exploration of MCMC Convergence in Bayesian Phylogenetic Inference. <http://king2.scs.fsu.edu/CEBProjects/awty/awty_start.php> (2004).
11 Nylander, J. A. A., Wilgenbusch, J. C., Warren, D. L. & Swofford, D. L. AWTY (Are we there yet?): a system for graphical exploration of MCMC convergence in Bayesian phylogenetics. *Bioinform.* **24**, 581-583, (2008).
12 Drummond, A. J. & Rambaut, A. BEAST: Bayesian evolutionary analysis by sampling trees. *BMC Evol. Biol.* **7**, 214, (2007).
13 Dornburg, A., Brandley, M. C., McGowen, M. R. & Near, T. J. Relaxed clocks and inferences of heterogeneous patterns of nucleotide substitution and divergence time estimates across whales and dolphins (Mammalia: Cetacea). *Mol. Biol. Evol.* **29**, 721-736, (2012).
14 Crisp, M., Hardy, N. & Cook, L. Clock model makes a large difference to age estimates of long-stemmed clades with no internal calibration: a test using Australian grasstrees. *BMC Evol. Biol.* **14**, 263, (2014).
15 Drummond, A. J. & Suchard, M. A. Bayesian random local clocks, or one rate to rule them all. *BMC biology* **8**, 114, (2010).
16 Drummond, A. J., Ho, S. Y. W., Phillips, M. J. & Rambaut, A. Relaxed phylogenetics and dating with confidence. *PLoS Biol.* **4**, e88, (2006).
17 Kass, R. E. & Raftery, A. E. Bayes factors and model uncertainty. (Carnegie Mellon University, Dept. of Statistics, 1993).
18 Kass, R. E. & Raftery, A. E. Bayes Factors. *J. Am. Stat. Assoc.* **90**, 773-795, (1995).
19 Baele, G. *et al.* Improving the accuracy of demographic and molecular clock model comparison while accommodating phylogenetic uncertainty. *Mol. Biol. Evol.* **29**, 2157-2167, (2012).
20 Baele, G., Li, W. L., Drummond, A. J., Suchard, M. A. & Lemey, P. Accurate model selection of relaxed molecular clocks in Bayesian phylogenetics. *Mol. Biol. Evol.*, (2012).
21 Christin, P. A. *et al.* Molecular dating, evolutionary rates, and the age of the grasses. *Syst. Biol.* **63**, 153-165, (2014).
22 Bouchenak-Khelladi, Y., Slingsby, J. A., Verboom, G. A. & Bond, W. J. Diversification of C4 grasses (Poaceae) does not coincide with their ecological dominance. *Am. J. Bot.* **101**, 300-307, (2014).
23 Prasad, V. *et al.* Late Cretaceous origin of the rice tribe provides evidence for early diversification in Poaceae. *Nature Commun.* **2**, 480, (2011).
24 Vicentini, A., Barber, J. C., Aliscioni, S. S., Giussani, L. M. & Kellogg, E. A. The age of the grasses and clusters of origins of C4 photosynthesis. *Glob. Change Biol.* **14**, 2963-2977, (2008).
25 Near, T. J. & Sanderson, M. J. Assessing the quality of molecular divergence time estimates by fossil calibrations and fossil-based model selection. *Philos. Trans. R. Soc. Lond. B Biol. Sci.* **359**, 1477-1483, (2004).
26 Stadler, T. On incomplete sampling under birth-death models and connections to the sampling-based coalescent. *J. Theor. Biol.* **261**, 58-66, (2009).

Supplementary data 3.1: Maximum likelihood (ML) phylogeny of Triodiinae and other Poaceae outgroups, derived using concatenated ITS-*matK* sequence data in RaxML. The tree is rooted with *Centropodia glauca* and *Merxmuellera rangei*. Bootstrap support is shown on branches. Terminals are coloured by position of leaf stomata: epistomatous taxa are green and amphistomatous are blue. Parsimony reconstruction of biome is shown at nodes: savannah is green and Eremaean is red.

Supplementary data 3.2: Bayesian maximum clade credibility (MCC) phylogeny of Triodiinae and other Poaceae outgroups, derived using concatenated ITS-*matK* sequence data in MrBayes. The tree is rooted with *Centropodia glauca* and *Merxmuellera rangei*. Posterior probability is shown on branches. Terminals are coloured by position of leaf stomata: epistomatous taxa are green and amphistomatous are blue. Parsimony reconstruction of biome is shown at nodes: savannah is green and Eremaean is red.

Supplementary data 3.3: Maximum likelihood (ML) phylogeny of Triodiinae and other Poaceae outgroups, derived using ITS sequences in RaxML. The tree is rooted with *Centropodia glauca* and *Merxmuellera rangei*. Solid circles on branches show ML bootstrap values ≥ 80 and posterior probabilities ≥ 0.95 (from MrBayes analysis). Open circles on branches show posterior probabilities ≥ 0.95. Terminals are coloured by position of leaf stomata: epistomatous taxa are green and amphistomatous are blue. Parsimony reconstruction of biome is shown at nodes: savannah is green and Eremaean is red.

Supplementary data 3.4: Maximum likelihood (ML) phylogeny of Triodiinae and other Poaceae outgroups, derived using *matK* sequences in RaxML. The tree is rooted with *Centropodia glauca* and *Merxmuellera rangei*. Solid circles on branches show ML bootstrap values ≥ 80 and posterior probabilities ≥ 0.95 (from MrBayes analysis). Open circles on branches show posterior probabilities ≥ 0.95. Terminals are coloured by position of leaf stomata: epistomatous taxa are green and amphistomatous are blue. Parsimony reconstruction of biome is shown at nodes: savannah is green and Eremaean is red.

Supplementary data 3.5: Chronogram of concatenated ITS and *matK* datasets showing the node ages and relationships within Triodiinae inferred using BEAST with a Random Local Clocks (RLC) model. Posterior probabilities are shown on nodes. Node bars indicate 95% highest posterior density of age estimates.

Supplementary data 3.6: Chronogram of concatenated ITS and *matK* datasets showing the node ages and relationships within Triodiinae inferred using BEAST with an Uncorrelated Lognormal (UCLN) model. Posterior probabilities are shown on nodes. Node bars indicate 95% highest posterior density of age estimates.

Supplementary data 3.7: Chronogram of Triodiinae estimated using ITS sequences in BEAST with a Random Local Clocks (RLC) model. Posterior probabilities are shown on nodes. Node bars indicate 95% highest posterior density of age estimates.

Supplementary data 3.8: Chronogram of Triodiinae estimated using *matK* sequences in BEAST with a Random Local Clocks (RLC) model. Posterior probabilities are shown on nodes. Node bars indicate 95% highest posterior density of age estimates.

Supplementary data 3.9: Set of credible diversification shifts in the posterior distribution of shifts estimated using BAMM.

Supplementary data 3.10: Proportion of Triodiinae leaf tissue identified as sclerenchyma from cross sections of amphistomatous and epistomatous species.

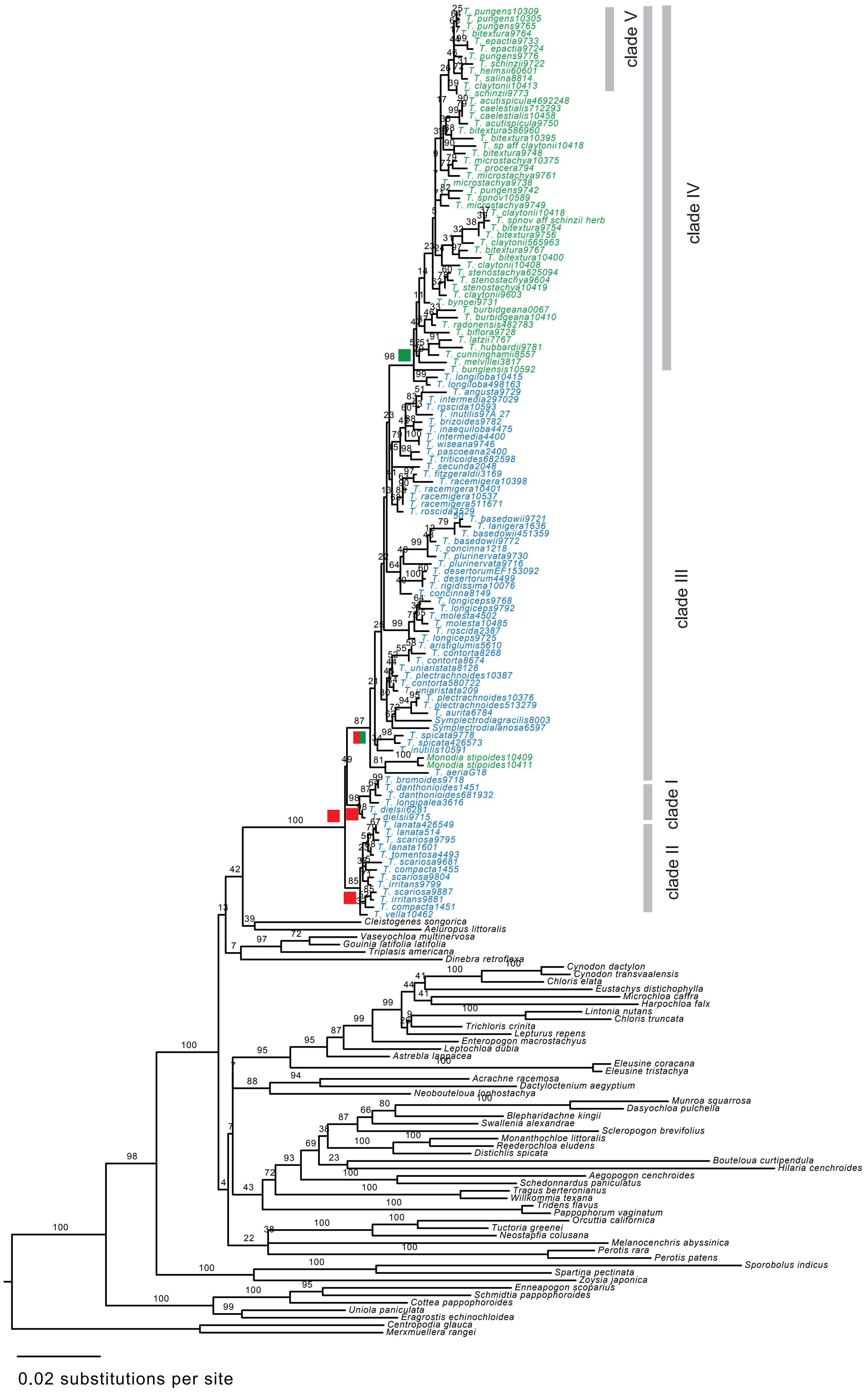

T. pungens10309
T. pungens10305
T. pungens9765
T. bitextura9764
T. epactia9733
T. epactia9724
T. pungens9776
T. schinzii9722
T. helmsii60601
T. salina8814
T. claytonii10413
T. schinzii9773
T. acutispicula4692248
T. caelestialis712293
T. caelestialis10458
T. acutispicula9750
T. bitextura586960
T. bitextura10395
T. sp aff claytonii10418
T. bitextura9748
T. microstachya10375
T. procera794
T. microstachya9761
T. microstachya9738
T. pungens9742
T. spnov10589
T. microstachya9749
T. claytonii10418
T. spnov aff schinzii herb
T. bitextura9754
T. bitextura9756
T. claytonii565963
T. bitextura9767
T. bitextura10400
T. claytonii10408
T. stenostachya625094
T. stenostachya9604
T. stenostachya10419
T. claytonii9603
T. bynoei9731
T. burbidgeana0067
T. burbidgeana10410
T. radonensis482783
T. biflora9728
T. latzii7767
T. hubbardii9781
T. cunninghamii8557
T. melvillei3817
T. bunglensis10592
T. longiloba10415
T. longiloba498163
T. angusta9729
T. intermedia297029
T. roscida10593
T. inutilis97A 27
T. brizoides9782
T. inaequiloba4475
T. intermedia4400
T. wiseana9746
T. pascoeana2400
T. triticoides682598
T. secunda2048
T. fitzgeraldii3169
T. racemigera10398
T. racemigera10401
T. racemigera10537
T. racemigera511671
T. roscida2529
T. basedowii9721
T. lanigera1636
T. basedowii451359
T. basedowii9772
T. concinna1218
T. plurinervata9730
T. plurinervata9716
T. desertorumEF153092
T. desertorum4499
T. rigidissima10076
T. concinna8149
T. longiceps9768
T. longiceps9792
T. molesta4502
T. molesta10485
T. roscida2387
T. longiceps9725
T. aristiglumis5610
T. contorta8268
T. contorta8674
T. uniaristata8128
T. plectrachnoides10387
T. contorta580722
T. uniaristata209
T. plectrachnoides10376
T. plectrachnoides513279
T. aurita6784
Symplectrodiagracilis8003
Symplectrodialanosa6597
T. spicata9778
T. spicata426573
T. inutilis10591
Monodia stipoides10409
Monodia stipoides10411
T. aeriaG18
T. bromoides9718
T. danthonioides1451
T. danthonioides681932
T. longipalea3616
T. dielsii6281
T. dielsii9715
T. lanata426549
T. lanata514
T. scariosa9795
T. lanata1601
T. tomentosa4493
T. scariosa9681
T. compacta1455
T. scariosa9804
T. irritans9799
T. scariosa9887
T. irritans9881
T. compacta1451
T. vella10462
Cleistogenes songorica
Aeluropus littoralis
Vaseyochloa multinervosa
Gouinia latifolia latifolia
Triplasis americana
Dinebra retroflexa
Cynodon dactylon
Cynodon transvaalensis
Chloris elata
Eustachys distichophylla
Microchloa caffra
Harpochloa falx
Lintonia nutans
Chloris truncata
Trichloris crinita
Lepturus repens
Enteropogon macrostachyus
Leptochloa dubia
Astrebla lappacea
Eleusine coracana
Eleusine tristachya
Acrachne racemosa
Dactyloctenium aegyptium
Neobouteloua lophostachya
Munroa squarrosa
Dasyochloa pulchella
Blepharidachne kingii
Swallenia alexandrae
Scleropogon brevifolius
Monanthochloe littoralis
Reederochloa eludens
Distichlis spicata
Bouteloua curtipendula
Hilaria cenchroides
Aegopogon cenchroides
Schedonnardus paniculatus
Tragus berteronianus
Willkommia texana
Tridens flavus
Pappophorum vaginatum
Orcuttia californica
Tuctoria greenei
Neostapfia colusana
Melanocenchris abyssinica
Perotis rara
Perotis patens
Sporobolus indicus
Spartina pectinata
Zoysia japonica
Enneapogon scoparius
Schmidtia pappophoroides
Cottea pappophoroides
Uniola paniculata
Eragrostis echinochloidea
Centropodia glauca
Merxmuellera rangei
clade V
clade IV
clade III
clade I
clade II
0.02 substitutions per site

Supp. data 3.1

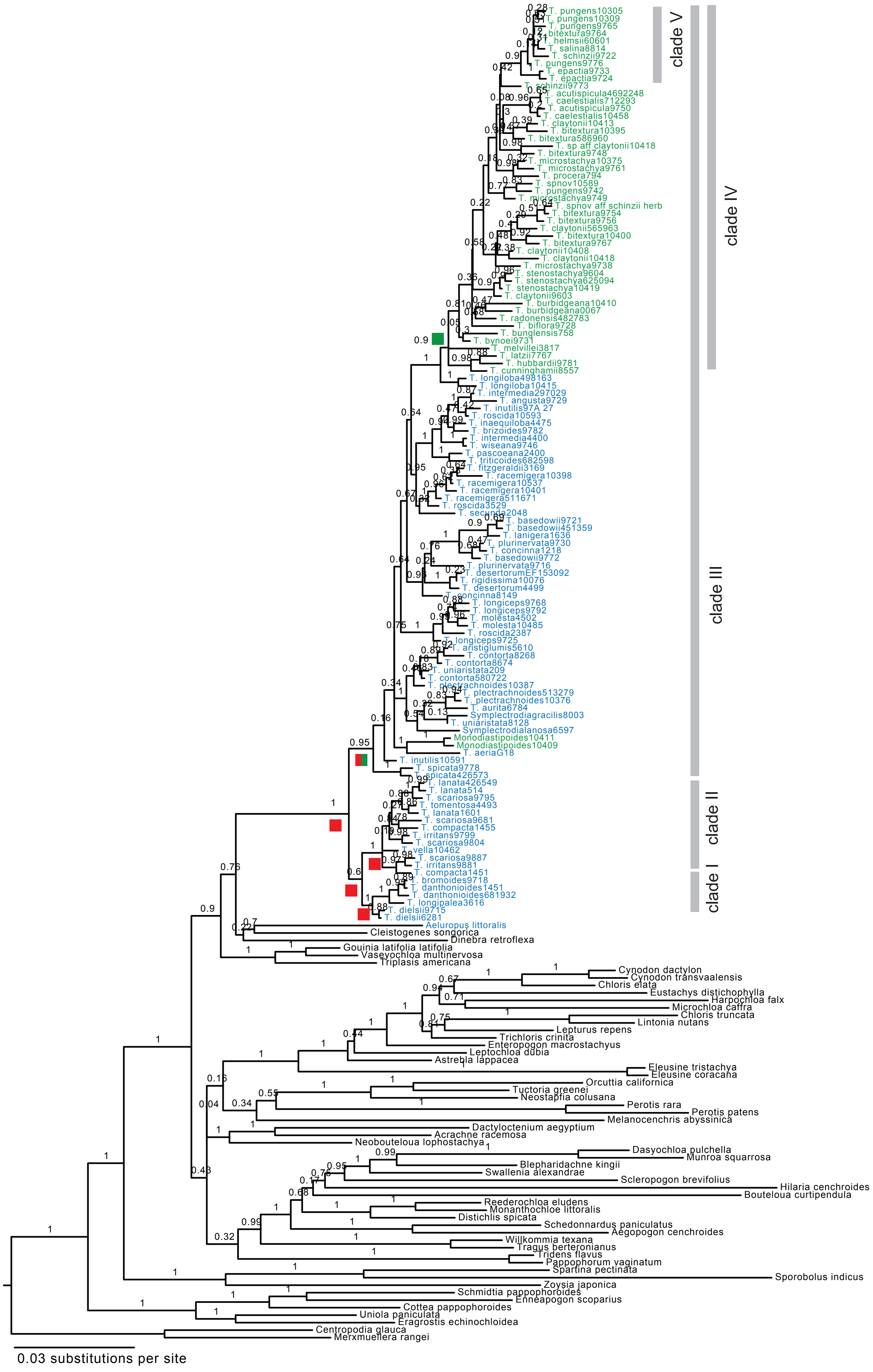
clade V
clade IV
clade III
clade II
clade I
T. pungens10305
T. pungens10309
T. pungens9765
T. bitextura9764
T. helmsii60601
T. salina8814
T. schinzii9722
T. pungens9776
T. epactia9733
T. epactia9724
T. schinzii9773
T. acutispicula4692248
T. caelestialis712293
T. acutispicula9750
T. caelestialis10458
T. claytonii10413
T. bitextura10395
T. bitextura586960
T. sp aff claytonii10418
T. bitextura9748
T. microstachya10375
T. microstachya9761
T. procera794
T. spnov10589
T. pungens9742
T. microstachya9749
T. spnov aff schinzii herb
T. bitextura9754
T. bitextura9756
T. claytonii565963
T. bitextura10400
T. bitextura9767
T. claytonii10408
T. claytonii10418
T. microstachya9738
T. stenostachya9604
T. stenostachya625094
T. stenostachya10419
T. claytonii9603
T. burbidgeana10410
T. burbidgeana0067
T. radonensis482783
T. biflora9728
T. bunglensis758
T. bynoei9731
T. melvillei3817
T. latzii7767
T. hubbardii9781
T. cunninghamii8557
T. longiloba498163
T. longiloba10415
T. intermedia297029
T. angusta9729
T. inutilis97A 27
T. roscida10593
T. inaequiloba4475
T. brizoides9782
T. intermedia4400
T. wiseana9746
T. pascoeana2400
T. triticoides682598
T. fitzgeraldii3169
T. racemigera10398
T. racemigera10537
T. racemigera10401
T. racemigera511671
T. roscida3529
T. secunda2048
T. basedowii9721
T. basedowii451359
T. lanigera1636
T. plurinervata9730
T. concinna1218
T. basedowii9772
T. plurinervata9716
T. desertorumEF153092
T. rigidissima10076
T. desertorum4499
T. concinna8149
T. longiceps9768
T. longiceps9792
T. molesta4502
T. molesta10485
T. roscida2387
T. longiceps9725
T. aristiglumis5610
T. contorta8268
T. contorta8674
T. uniaristata209
T. contorta580722
T. plectrachnoides10387
T. plectrachnoides513279
T. plectrachnoides10376
T. aurita6784
Symplectrodiagracilis8003
T. uniaristata8128
Symplectrodialanosa6597
Monodiastipoides10411
Monodiastipoides10409
T. aeriaG18
T. inutilis10591
T. spicata9778
T. spicata426573
T. lanata426549
T. lanata514
T. scariosa9795
T. tomentosa4493
T. lanata1601
T. scariosa9681
T. compacta1455
T. irritans9799
T. scariosa9804
T. vella10462
T. scariosa9887
T. irritans9881
T. compacta1451
T. bromoides9718
T. danthonioides1451
T. danthonioides681932
T. longipalea3616
T. dielsii9715
T. dielsii6281
Aeluropus littoralis
Cleistogenes songorica
Dinebra retroflexa
Gouinia latifolia latifolia
Vaseyochloa multinervosa
Triplasis americana
Cynodon dactylon
Cynodon transvaalensis
Chloris elata
Eustachys distichophylla
Harpochloa falx
Microchloa caffra
Chloris truncata
Lintonia nutans
Lepturus repens
Trichloris crinita
Enteropogon macrostachyus
Leptochloa dubia
Astrebla lappacea
Eleusine tristachya
Eleusine coracana
Orcuttia californica
Tuctoria greenei
Neostapfia colusana
Perotis rara
Perotis patens
Melanocenchris abyssinica
Dactyloctenium aegyptium
Acrachne racemosa
Neobouteloua lophostachya
Dasyochloa pulchella
Munroa squarrosa
Blepharidachne kingii
Swallenia alexandrae
Scleropogon brevifolius
Hilaria cenchroides
Bouteloua curtipendula
Reederochloa eludens
Monanthochloe littoralis
Distichlis spicata
Schedonnardus paniculatus
Aegopogon cenchroides
Willkommia texana
Tragus berteronianus
Tridens flavus
Pappophorum vaginatum
Spartina pectinata
Sporobolus indicus
Zoysia japonica
Schmidtia pappophoroides
Enneapogon scoparius
Cottea pappophoroides
Uniola paniculata
Eragrostis echinochloidea
Centropodia glauca
Merxmuellera rangei
0.03 substitutions per site

Supp. data 3.2

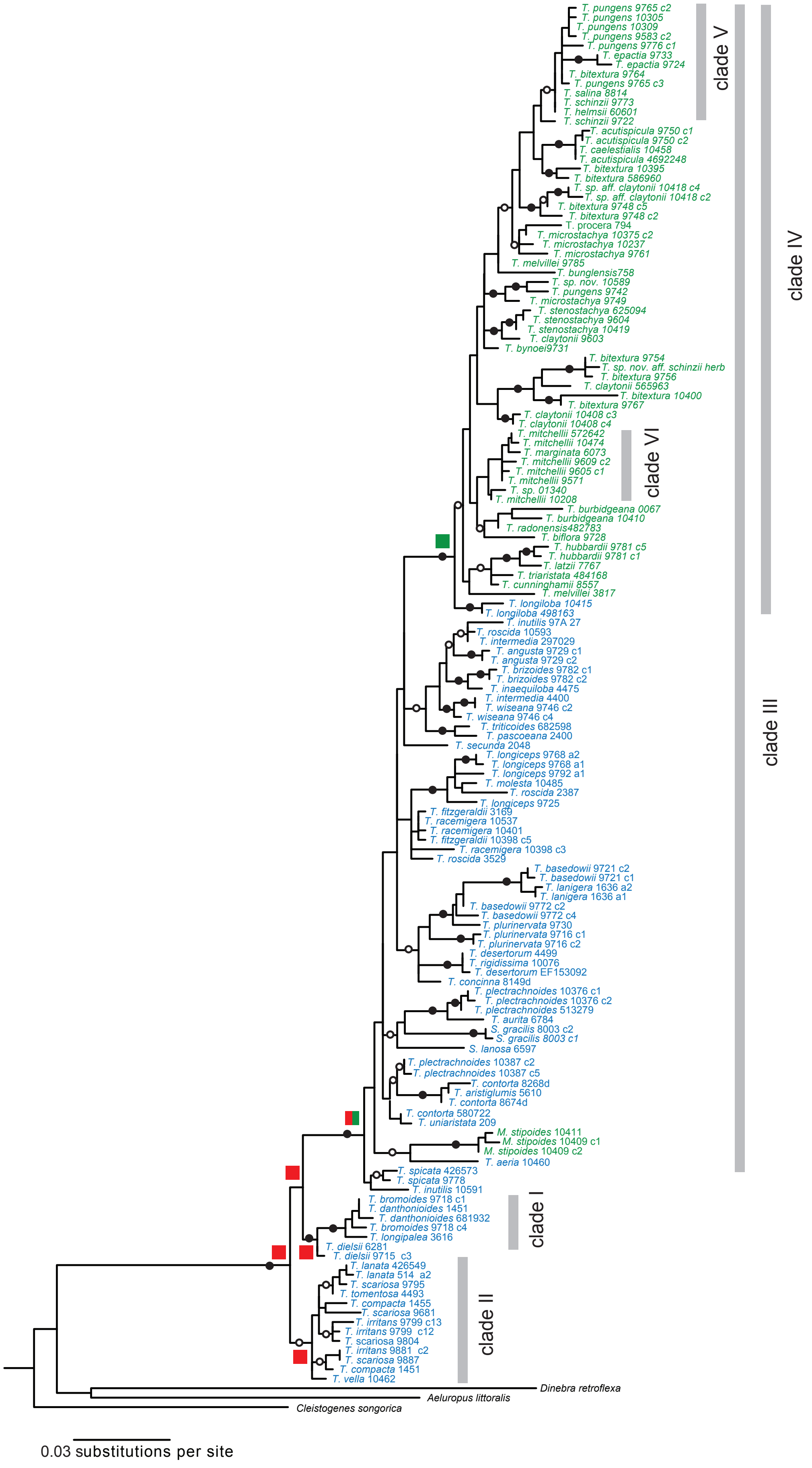
T. pungens 9765 c2
T. pungens 10305
T. pungens 10309
T. pungens 9583 c2
T. pungens 9776 c1
T. epactia 9733
T. epactia 9724
T. bitextura 9764
T. pungens 9765 c3
T. salina 8814
T. schinzii 9773
T. helmsii 60601
T. schinzii 9722
T. acutispicula 9750 c1
T. acutispicula 9750 c2
T. caelestialis 10458
T. acutispicula 4692248
T. bitextura 10395
T. bitextura 586960
T. sp. aff. claytonii 10418 c4
T. sp. aff. claytonii 10418 c2
T. bitextura 9748 c5
T. bitextura 9748 c2
T. procera 794
T. microstachya 10375 c2
T. microstachya 10237
T. microstachya 9761
T. melvillei 9785
T. bunglensis758
T. sp. nov. 10589
T. pungens 9742
T. microstachya 9749
T. stenostachya 625094
T. stenostachya 9604
T. stenostachya 10419
T. claytonii 9603
T. bynoei9731
T. bitextura 9754
T. sp. nov. aff. schinzii herb
T. bitextura 9756
T. claytonii 565963
T. bitextura 10400
T. bitextura 9767
T. claytonii 10408 c3
T. claytonii 10408 c4
T. mitchellii 572642
T. mitchellii 10474
T. marginata 6073
T. mitchellii 9609 c2
T. mitchellii 9605 c1
T. mitchellii 9571
T. sp. 01340
T. mitchellii 10208
T. burbidgeana 0067
T. burbidgeana 10410
T. radonensis482783
T. biflora 9728
T. hubbardii 9781 c5
T. hubbardii 9781 c1
T. latzii 7767
T. triaristata 484168
T. cunninghamii 8557
T. melvillei 3817
T. longiloba 10415
T. longiloba 498163
T. inutilis 97A 27
T. roscida 10593
T. intermedia 297029
T. angusta 9729 c1
T. angusta 9729 c2
T. brizoides 9782 c1
T. brizoides 9782 c2
T. inaequiloba 4475
T. intermedia 4400
T. wiseana 9746 c2
T. wiseana 9746 c4
T. triticoides 682598
T. pascoeana 2400
T. secunda 2048
T. longiceps 9768 a2
T. longiceps 9768 a1
T. longiceps 9792 a1
T. molesta 10485
T. roscida 2387
T. longiceps 9725
T. fitzgeraldii 3169
T. racemigera 10537
T. racemigera 10401
T. fitzgeraldii 10398 c5
T. racemigera 10398 c3
T. roscida 3529
T. basedowii 9721 c2
T. basedowii 9721 c1
T. lanigera 1636 a2
T. lanigera 1636 a1
T. basedowii 9772 c2
T. basedowii 9772 c4
T. plurinervata 9730
T. plurinervata 9716 c1
T. plurinervata 9716 c2
T. desertorum 4499
T. rigidissima 10076
T. desertorum EF153092
T. concinna 8149d
T. plectrachnoides 10376 c1
T. plectrachnoides 10376 c2
T. plectrachnoides 513279
T. aurita 6784
S. gracilis 8003 c2
S. gracilis 8003 c1
S. lanosa 6597
T. plectrachnoides 10387 c2
T. plectrachnoides 10387 c5
T. contorta 8268d
T. aristiglumis 5610
T. contorta 8674d
T. contorta 580722
T. uniaristata 209
M. stipoides 10411
M. stipoides 10409 c1
M. stipoides 10409 c2
T. aeria 10460
T. spicata 426573
T. spicata 9778
T. inutilis 10591
T. bromoides 9718 c1
T. danthonioides 1451
T. danthonioides 681932
T. bromoides 9718 c4
T. longipalea 3616
T. dielsii 6281
T. dielsii 9715 c3
T. lanata 426549
T. lanata 514 a2
T. scariosa 9795
T. tomentosa 4493
T. compacta 1455
T. scariosa 9681
T. irritans 9799 c13
T. irritans 9799 c12
T. scariosa 9804
T. irritans 9881 c2
T. scariosa 9887
T. compacta 1451
T. vella 10462
Dinebra retroflexa
Aeluropus littoralis
Cleistogenes songorica
clade V
clade VI
clade IV
clade III
clade I
clade II
0.03 substitutions per site

Supp. data 3.3

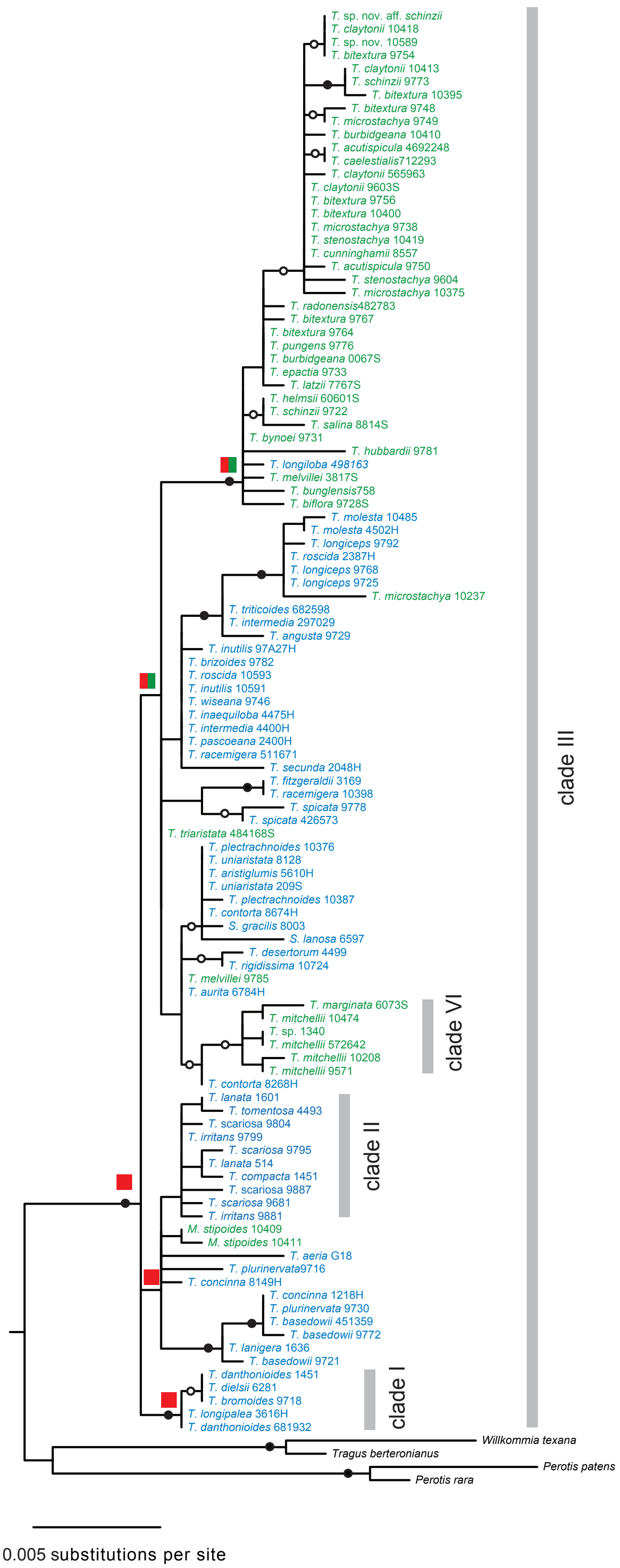
T. sp. nov. aff. schinzii
T. claytonii 10418
T. sp. nov. 10589
T. bitextura 9754
T. claytonii 10413
T. schinzii 9773
T. bitextura 10395
T. bitextura 9748
T. microstachya 9749
T. burbidgeana 10410
T. acutispicula 4692248
T. caelestialis712293
T. claytonii 565963
T. claytonii 9603S
T. bitextura 9756
T. bitextura 10400
T. microstachya 9738
T. stenostachya 10419
T. cunninghamii 8557
T. acutispicula 9750
T. stenostachya 9604
T. microstachya 10375
T. radonensis482783
T. bitextura 9767
T. bitextura 9764
T. pungens 9776
T. burbidgeana 0067S
T. epactia 9733
T. latzii 7767S
T. helmsii 60601S
T. schinzii 9722
T. salina 8814S
T. bynoei 9731
T. hubbardii 9781
T. longiloba 498163
T. melvillei 3817S
T. bunglensis758
T. biflora 9728S
T. molesta 10485
T. molesta 4502H
T. longiceps 9792
T. roscida 2387H
T. longiceps 9768
T. longiceps 9725
T. microstachya 10237
T. triticoides 682598
T. intermedia 297029
T. angusta 9729
T. inutilis 97A27H
T. brizoides 9782
T. roscida 10593
T. inutilis 10591
T. wiseana 9746
T. inaequiloba 4475H
T. intermedia 4400H
T. pascoeana 2400H
T. racemigera 511671
T. secunda 2048H
T. fitzgeraldii 3169
T. racemigera 10398
T. spicata 9778
T. spicata 426573
T. triaristata 484168S
T. plectrachnoides 10376
T. uniaristata 8128
T. aristiglumis 5610H
T. uniaristata 209S
T. plectrachnoides 10387
T. contorta 8674H
S. gracilis 8003
S. lanosa 6597
T. desertorum 4499
T. rigidissima 10724
T. melvillei 9785
T. aurita 6784H
T. marginata 6073S
T. mitchellii 10474
T. sp. 1340
T. mitchellii 572642
T. mitchellii 10208
T. mitchellii 9571
T. contorta 8268H
T. lanata 1601
T. tomentosa 4493
T. scariosa 9804
T. irritans 9799
T. scariosa 9795
T. lanata 514
T. compacta 1451
T. scariosa 9887
T. scariosa 9681
T. irritans 9881
M. stipoides 10409
M. stipoides 10411
T. aeria G18
T. plurinervata9716
T. concinna 8149H
T. concinna 1218H
T. plurinervata 9730
T. basedowii 451359
T. basedowii 9772
T. lanigera 1636
T. basedowii 9721
T. danthonioides 1451
T. dielsii 6281
T. bromoides 9718
T. longipalea 3616H
T. danthonioides 681932
Willkommia texana
Tragus berteronianus
Perotis patens
Perotis rara
clade III
clade VI
clade II
clade I
0.005 substitutions per site

Supp. data 3.4

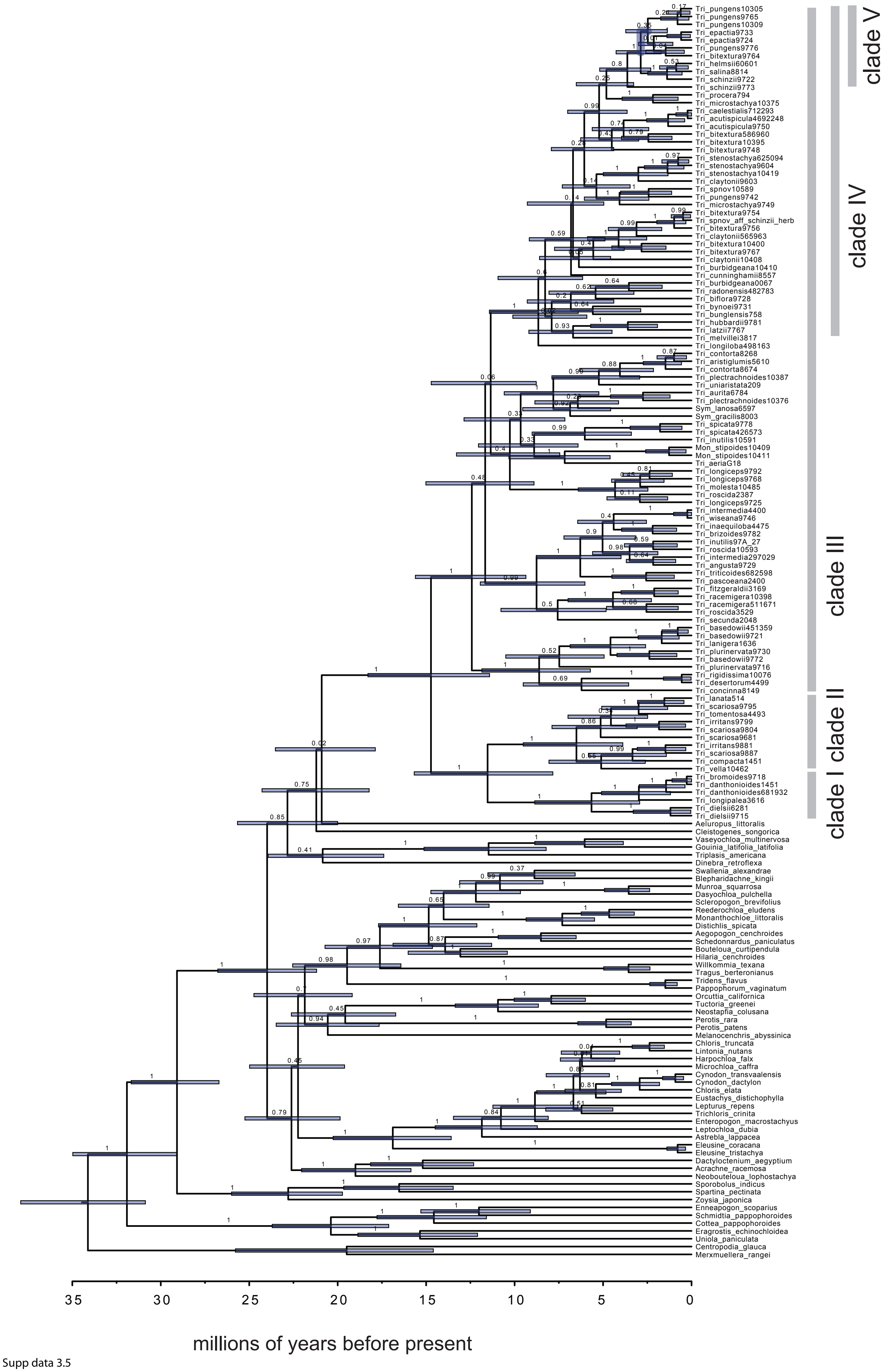
Tri_pungens10305
Tri_pungens9765
Tri_pungens10309
Tri_epactia9733
Tri_epactia9724
Tri_pungens9776
Tri_bitextura9764
Tri_helmsii60601
Tri_salina8814
Tri_schinzii9722
Tri_schinzii9773
Tri_procera794
Tri_microstachya10375
Tri_caelestialis712293
Tri_acutispicula4692248
Tri_acutispicula9750
Tri_bitextura586960
Tri_bitextura10395
Tri_bitextura9748
Tri_stenostachya625094
Tri_stenostachya9604
Tri_stenostachya10419
Tri_claytonii9603
Tri_spnov10589
Tri_pungens9742
Tri_microstachya9749
Tri_bitextura9754
Tri_spnov_aff_schinzii_herb
Tri_bitextura9756
Tri_claytonii565963
Tri_bitextura10400
Tri_bitextura9767
Tri_claytonii10408
Tri_burbidgeana10410
Tri_cunninghamii8557
Tri_burbidgeana0067
Tri_radonensis482783
Tri_biflora9728
Tri_bynoei9731
Tri_bunglensis758
Tri_hubbardii9781
Tri_latzii7767
Tri_melvillei3817
Tri_longiloba498163
Tri_contorta8268
Tri_aristiglumis5610
Tri_contorta8674
Tri_plectrachnoides10387
Tri_uniaristata209
Tri_aurita6784
Tri_plectrachnoides10376
Sym_lanosa6597
Sym_gracilis8003
Tri_spicata9778
Tri_spicata426573
Tri_inutilis10591
Mon_stipoides10409
Mon_stipoides10411
Tri_aeriaG18
Tri_longiceps9792
Tri_longiceps9768
Tri_molesta10485
Tri_roscida2387
Tri_longiceps9725
Tri_intermedia4400
Tri_wiseana9746
Tri_inaequiloba4475
Tri_brizoides9782
Tri_inutilis97A_27
Tri_roscida10593
Tri_intermedia297029
Tri_angusta9729
Tri_triticoides682598
Tri_pascoeana2400
Tri_fitzgeraldii3169
Tri_racemigera10398
Tri_racemigera511671
Tri_roscida3529
Tri_secunda2048
Tri_basedowii451359
Tri_basedowii9721
Tri_lanigera1636
Tri_plurinervata9730
Tri_basedowii9772
Tri_plurinervata9716
Tri_rigidissima10076
Tri_desertorum4499
Tri_concinna8149
Tri_lanata514
Tri_scariosa9795
Tri_tomentosa4493
Tri_irritans9799
Tri_scariosa9804
Tri_scariosa9681
Tri_irritans9881
Tri_scariosa9887
Tri_compacta1451
Tri_vella10462
Tri_bromoides9718
Tri_danthonioides1451
Tri_danthonioides681932
Tri_longipalea3616
Tri_dielsii6281
Tri_dielsii9715
Aeluropus_littoralis
Cleistogenes_songorica
Vaseyochloa_multinervosa
Gouinia_latifolia_latifolia
Triplasis_americana
Dinebra_retroflexa
Swallenia_alexandrae
Blepharidachne_kingii
Munroa_squarrosa
Dasyochloa_pulchella
Scleropogon_brevifolius
Reederochloa_eludens
Monanthochloe_littoralis
Distichlis_spicata
Aegopogon_cenchroides
Schedonnardus_paniculatus
Bouteloua_curtipendula
Hilaria_cenchroides
Willkommia_texana
Tragus_berteronianus
Tridens_flavus
Pappophorum_vaginatum
Orcuttia_californica
Tuctoria_greenei
Neostapfia_colusana
Perotis_rara
Perotis_patens
Melanocenchris_abyssinica
Chloris_truncata
Lintonia_nutans
Harpochloa_falx
Microchloa_caffra
Cynodon_transvaalensis
Cynodon_dactylon
Chloris_elata
Eustachys_distichophylla
Lepturus_repens
Trichloris_crinita
Enteropogon_macrostachyus
Leptochloa_dubia
Astrebla_lappacea
Eleusine_coracana
Eleusine_tristachya
Dactyloctenium_aegyptium
Acrachne_racemosa
Neobouteloua_lophostachya
Sporobolus_indicus
Spartina_pectinata
Zoysia_japonica
Enneapogon_scoparius
Schmidtia_pappophoroides
Cottea_pappophoroides
Eragrostis_echinochloidea
Uniola_paniculata
Centropodia_glauca
Merxmuellera_rangei
clade V
clade IV
clade III
clade II
clade I
35
30
25
20
15
10
5
0
millions of years before present

Supp data 3.5

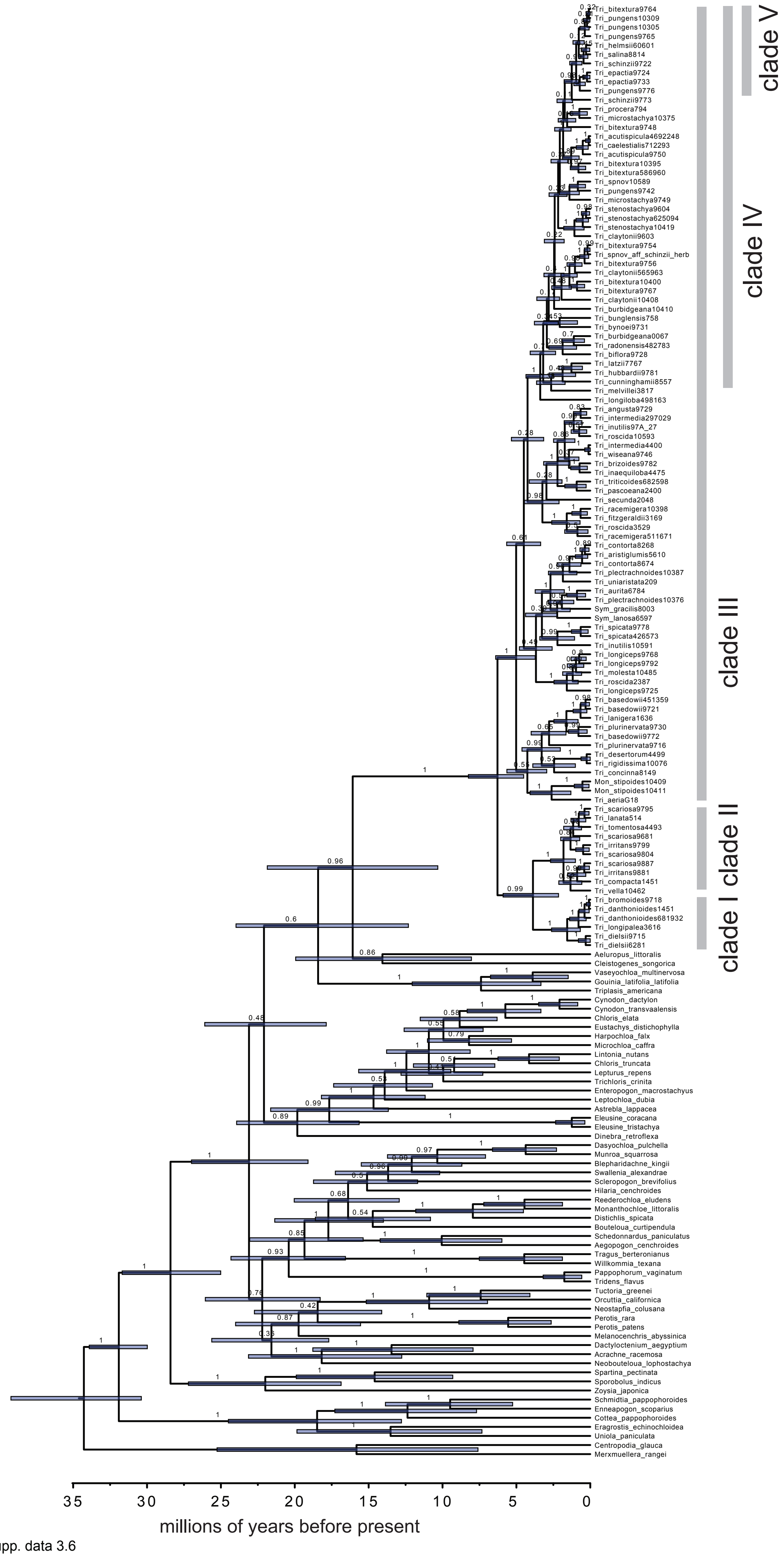

Tri_bitextura9764
Tri_pungens10309
Tri_pungens10305
Tri_pungens9765
Tri_helmsii60601
Tri_salina8814
Tri_schinzii9722
Tri_epactia9724
Tri_epactia9733
Tri_pungens9776
Tri_schinzii9773
Tri_procera794
Tri_microstachya10375
Tri_bitextura9748
Tri_acutispicula4692248
Tri_caelestialis712293
Tri_acutispicula9750
Tri_bitextura10395
Tri_bitextura586960
Tri_spnov10589
Tri_pungens9742
Tri_microstachya9749
Tri_stenostachya9604
Tri_stenostachya625094
Tri_stenostachya10419
Tri_claytonii9603
Tri_bitextura9754
Tri_spnov_aff_schinzii_herb
Tri_bitextura9756
Tri_claytonii565963
Tri_bitextura10400
Tri_bitextura9767
Tri_claytonii10408
Tri_burbidgeana10410
Tri_bunglensis758
Tri_bynoei9731
Tri_burbidgeana0067
Tri_radonensis482783
Tri_biflora9728
Tri_latzii7767
Tri_hubbardii9781
Tri_cunninghamii8557
Tri_melvillei3817
Tri_longiloba498163
Tri_angusta9729
Tri_intermedia297029
Tri_inutilis97A_27
Tri_roscida10593
Tri_intermedia4400
Tri_wiseana9746
Tri_brizoides9782
Tri_inaequiloba4475
Tri_triticoides682598
Tri_pascoeana2400
Tri_secunda2048
Tri_racemigera10398
Tri_fitzgeraldii3169
Tri_roscida3529
Tri_racemigera511671
Tri_contorta8268
Tri_aristiglumis5610
Tri_contorta8674
Tri_plectrachnoides10387
Tri_uniaristata209
Tri_aurita6784
Tri_plectrachnoides10376
Sym_gracilis8003
Sym_lanosa6597
Tri_spicata9778
Tri_spicata426573
Tri_inutilis10591
Tri_longiceps9768
Tri_longiceps9792
Tri_molesta10485
Tri_roscida2387
Tri_longiceps9725
Tri_basedowii451359
Tri_basedowii9721
Tri_lanigera1636
Tri_plurinervata9730
Tri_basedowii9772
Tri_plurinervata9716
Tri_desertorum4499
Tri_rigidissima10076
Tri_concinna8149
Mon_stipoides10409
Mon_stipoides10411
Tri_aeriaG18
Tri_scariosa9795
Tri_lanata514
Tri_tomentosa4493
Tri_scariosa9681
Tri_irritans9799
Tri_scariosa9804
Tri_scariosa9887
Tri_irritans9881
Tri_compacta1451
Tri_vella10462
Tri_bromoides9718
Tri_danthonioides1451
Tri_danthonioides681932
Tri_longipalea3616
Tri_dielsii9715
Tri_dielsii6281
Aeluropus_littoralis
Cleistogenes_songorica
Vaseyochloa_multinervosa
Gouinia_latifolia_latifolia
Triplasis_americana
Cynodon_dactylon
Cynodon_transvaalensis
Chloris_elata
Eustachys_distichophylla
Harpochloa_falx
Microchloa_caffra
Lintonia_nutans
Chloris_truncata
Lepturus_repens
Trichloris_crinita
Enteropogon_macrostachyus
Leptochloa_dubia
Astrebla_lappacea
Eleusine_coracana
Eleusine_tristachya
Dinebra_retroflexa
Dasyochloa_pulchella
Munroa_squarrosa
Blepharidachne_kingii
Swallenia_alexandrae
Scleropogon_brevifolius
Hilaria_cenchroides
Reederochloa_eludens
Monanthochloe_littoralis
Distichlis_spicata
Bouteloua_curtipendula
Schedonnardus_paniculatus
Aegopogon_cenchroides
Tragus_berteronianus
Willkommia_texana
Pappophorum_vaginatum
Tridens_flavus
Tuctoria_greenei
Orcuttia_californica
Neostapfia_colusana
Perotis_rara
Perotis_patens
Melanocenchris_abyssinica
Dactyloctenium_aegyptium
Acrachne_racemosa
Neobouteloua_lophostachya
Spartina_pectinata
Sporobolus_indicus
Zoysia_japonica
Schmidtia_pappophoroides
Enneapogon_scoparius
Cottea_pappophoroides
Eragrostis_echinochloidea
Uniola_paniculata
Centropodia_glauca
Merxmuellera_rangei
clade V
clade IV
clade III
clade II
clade I
35
30
25
20
15
10
5
0
millions of years before present

Supp. data 3.6

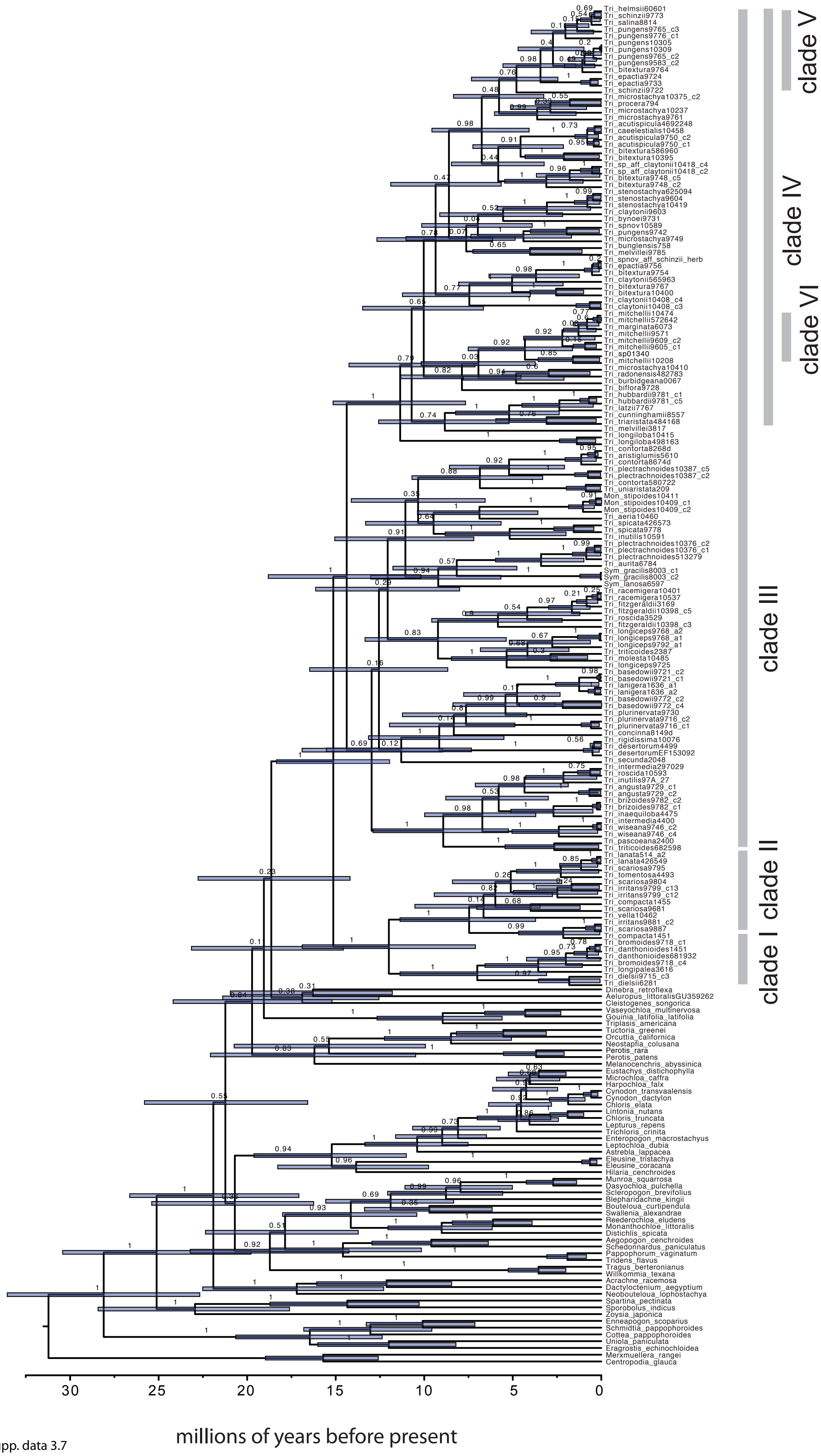
Tri_helmsii60601
Tri_schinzii9773
Tri_salina8814
Tri_pungens9765_c3
Tri_pungens9776_c1
Tri_pungens10305
Tri_pungens10309
Tri_pungens9765_c2
Tri_pungens9583_c2
Tri_bitextura9764
Tri_epactia9724
Tri_epactia9733
Tri_schinzii9722
Tri_microstachya10375_c2
Tri_procera794
Tri_microstachya10237
Tri_microstachya9761
Tri_acutispicula4692248
Tri_caeelestialis10458
Tri_acutispicula9750_c2
Tri_acutispicula9750_c1
Tri_bitextura586960
Tri_bitextura10395
Tri_sp_aff_claytonii10418_c4
Tri_sp_aff_claytonii10418_c2
Tri_bitextura9748_c5
Tri_bitextura9748_c2
Tri_stenostachya625094
Tri_stenostachya9604
Tri_stenostachya10419
Tri_claytonii9603
Tri_bynoei9731
Tri_spnov10589
Tri_pungens9742
Tri_microstachya9749
Tri_bunglensis758
Tri_melvillei9785
Tri_spnov_aff_schinzii_herb
Tri_epactia9756
Tri_bitextura9754
Tri_claytonii565963
Tri_bitextura9767
Tri_bitextura10400
Tri_claytonii10408_c4
Tri_claytonii10408_c3
Tri_mitchellii10474
Tri_mitchellii572642
Tri_marginata6073
Tri_mitchellii9571
Tri_mitchellii9609_c2
Tri_mitchellii9605_c1
Tri_sp01340
Tri_mitchellii10208
Tri_microstachya10410
Tri_radonensis482783
Tri_burbidgeana0067
Tri_biflora9728
Tri_hubbardii9781_c1
Tri_hubbardii9781_c5
Tri_latzii7767
Tri_cunninghamii8557
Tri_triaristata484168
Tri_melvillei3817
Tri_longiloba10415
Tri_longiloba498163
Tri_contorta8268d
Tri_aristiglumis5610
Tri_contorta8674d
Tri_plectrachnoides10387_c5
Tri_plectrachnoides10387_c2
Tri_contorta580722
Tri_uniaristata209
Mon_stipoides10411
Mon_stipoides10409_c1
Mon_stipoides10409_c2
Tri_aeria10460
Tri_spicata426573
Tri_spicata9778
Tri_inutilis10591
Tri_plectrachnoides10376_c2
Tri_plectrachnoides10376_c1
Tri_plectrachnoides513279
Tri_aurita6784
Sym_gracilis8003_c1
Sym_gracilis8003_c2
Sym_lanosa6597
Tri_racemigera10401
Tri_racemigera10537
Tri_fitzgeraldii3169
Tri_fitzgeraldii10398_c5
Tri_roscida3529
Tri_fitzgeraldii10398_c3
Tri_longiceps9768_a2
Tri_longiceps9768_a1
Tri_longiceps9792_a1
Tri_triticoides2387
Tri_molesta10485
Tri_longiceps9725
Tri_basedowii9721_c2
Tri_basedowii9721_c1
Tri_lanigera1636_a1
Tri_lanigera1636_a2
Tri_basedowii9772_c2
Tri_basedowii9772_c4
Tri_plurinervata9730
Tri_plurinervata9716_c2
Tri_plurinervata9716_c1
Tri_concinna8149d
Tri_rigidissima10076
Tri_desertorum4499
Tri_desertorumEF153092
Tri_secunda2048
Tri_intermedia297029
Tri_roscida10593
Tri_inutilis97A_27
Tri_angusta9729_c1
Tri_angusta9729_c2
Tri_brizoides9782_c2
Tri_brizoides9782_c1
Tri_inaequiloba4475
Tri_intermedia4400
Tri_wiseana9746_c2
Tri_wiseana9746_c4
Tri_pascoeana2400
Tri_triticoides682598
Tri_lanata514_a2
Tri_lanata426549
Tri_scariosa9795
Tri_tomentosa4493
Tri_scariosa9804
Tri_irritans9799_c13
Tri_irritans9799_c12
Tri_compacta1455
Tri_scariosa9681
Tri_vella10462
Tri_irritans9881_c2
Tri_scariosa9887
Tri_compacta1451
Tri_bromoides9718_c1
Tri_danthonioides1451
Tri_danthonioides681932
Tri_bromoides9718_c4
Tri_longipalea3616
Tri_dielsii9715_c3
Tri_dielsii6281
Dinebra_retroflexa
Aeluropus_littoralisGU359262
Cleistogenes_songorica
Vaseyochloa_multinervosa
Gouinia_latifolia_latifolia
Triplasis_americana
Tuctoria_greenei
Orcuttia_californica
Neostapfia_colusana
Perotis_rara
Perotis_patens
Melanocenchris_abyssinica
Eustachys_distichophylla
Microchloa_caffra
Harpochloa_falx
Cynodon_transvaalensis
Cynodon_dactylon
Chloris_elata
Lintonia_nutans
Chloris_truncata
Lepturus_repens
Trichloris_crinita
Enteropogon_macrostachyus
Leptochloa_dubia
Astrebla_lappacea
Eleusine_tristachya
Eleusine_coracana
Hilaria_cenchroides
Munroa_squarrosa
Dasyochloa_pulchella
Scleropogon_brevifolius
Blepharidachne_kingii
Bouteloua_curtipendula
Swallenia_alexandrae
Reederochloa_eludens
Monanthochloe_littoralis
Distichlis_spicata
Aegopogon_cenchroides
Schedonnardus_paniculatus
Pappophorum_vaginatum
Tridens_flavus
Tragus_berteronianus
Willkommia_texana
Acrachne_racemosa
Dactyloctenium_aegyptium
Neobouteloua_lophostachya
Spartina_pectinata
Sporobolus_indicus
Zoysia_japonica
Enneapogon_scoparius
Schmidtia_pappophoroides
Cottea_pappophoroides
Uniola_paniculata
Eragrostis_echinochloidea
Merxmuellera_rangei
Centropodia_glauca
clade V
clade IV
clade VI
clade III
clade II
clade I
30
25
20
15
10
5
0
millions of years before present

Supp. data 3.7

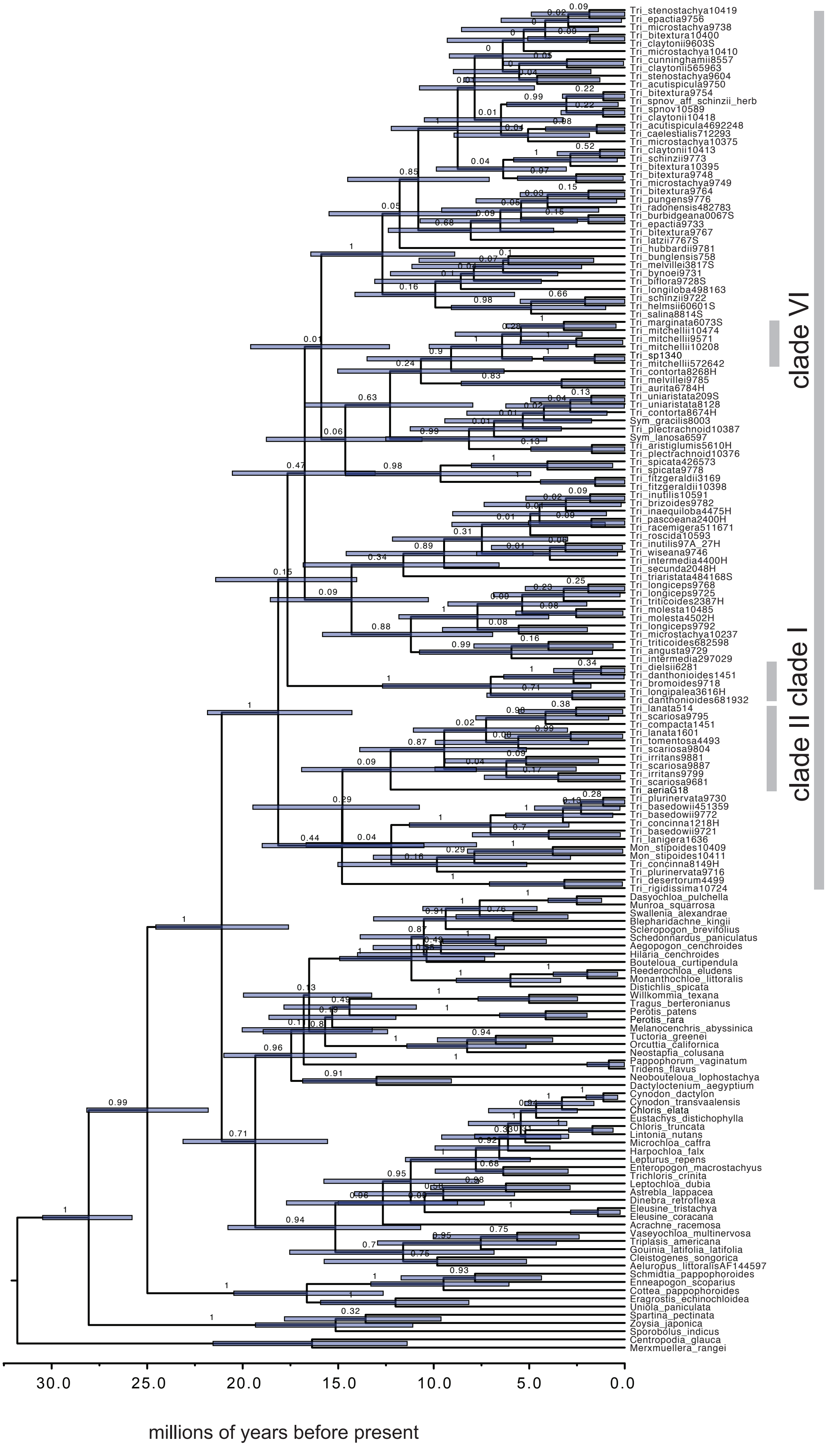
Tri_stenostachya10419
Tri_epactia9756
Tri_microstachya9738
Tri_bitextura10400
Tri_claytonii9603S
Tri_microstachya10410
Tri_cunninghamii8557
Tri_claytonii565963
Tri_stenostachya9604
Tri_acutispicula9750
Tri_bitextura9754
Tri_spnov_aff_schinzii_herb
Tri_spnov10589
Tri_claytonii10418
Tri_acutispicula4692248
Tri_caelestialis712293
Tri_microstachya10375
Tri_claytonii10413
Tri_schinzii9773
Tri_bitextura10395
Tri_bitextura9748
Tri_microstachya9749
Tri_bitextura9764
Tri_pungens9776
Tri_radonensis482783
Tri_burbidgeana0067S
Tri_epactia9733
Tri_bitextura9767
Tri_latzii7767S
Tri_hubbardii9781
Tri_bunglensis758
Tri_melvillei3817S
Tri_bynoei9731
Tri_biflora9728S
Tri_longiloba498163
Tri_schinzii9722
Tri_helmsii60601S
Tri_salina8814S
Tri_marginata6073S
Tri_mitchellii10474
Tri_mitchellii9571
Tri_mitchellii10208
Tri_sp1340
Tri_mitchellii572642
Tri_contorta8268H
Tri_melvillei9785
Tri_aurita6784H
Tri_uniaristata209S
Tri_uniaristata8128
Tri_contorta8674H
Sym_gracilis8003
Tri_plectrachnoid10387
Sym_lanosa6597
Tri_aristiglumis5610H
Tri_plectrachnoid10376
Tri_spicata426573
Tri_spicata9778
Tri_fitzgeraldii3169
Tri_fitzgeraldii10398
Tri_inutilis10591
Tri_brizoides9782
Tri_inaequiloba4475H
Tri_pascoeana2400H
Tri_racemigera511671
Tri_roscida10593
Tri_inutilis97A_27H
Tri_wiseana9746
Tri_intermedia4400H
Tri_secunda2048H
Tri_triaristata484168S
Tri_longiceps9768
Tri_longiceps9725
Tri_triticoides2387H
Tri_molesta10485
Tri_molesta4502H
Tri_longiceps9792
Tri_microstachya10237
Tri_triticoides682598
Tri_angusta9729
Tri_intermedia297029
Tri_dielsii6281
Tri_danthonioides1451
Tri_bromoides9718
Tri_longipalea3616H
Tri_danthonioides681932
Tri_lanata514
Tri_scariosa9795
Tri_compacta1451
Tri_lanata1601
Tri_tomentosa4493
Tri_scariosa9804
Tri_irritans9881
Tri_scariosa9887
Tri_irritans9799
Tri_scariosa9681
Tri_aeriaG18
Tri_plurinervata9730
Tri_basedowii451359
Tri_basedowii9772
Tri_concinna1218H
Tri_basedowii9721
Tri_lanigera1636
Mon_stipoides10409
Mon_stipoides10411
Tri_concinna8149H
Tri_plurinervata9716
Tri_desertorum4499
Tri_rigidissima10724
Dasyochloa_pulchella
Munroa_squarrosa
Swallenia_alexandrae
Blepharidachne_kingii
Scleropogon_brevifolius
Schedonnardus_paniculatus
Aegopogon_cenchroides
Hilaria_cenchroides
Bouteloua_curtipendula
Reederochloa_eludens
Monanthochloe_littoralis
Distichlis_spicata
Willkommia_texana
Tragus_berteronianus
Perotis_patens
Perotis_rara
Melanocenchris_abyssinica
Tuctoria_greenei
Orcuttia_californica
Neostapfia_colusana
Pappophorum_vaginatum
Tridens_flavus
Neobouteloua_lophostachya
Dactyloctenium_aegyptium
Cynodon_dactylon
Cynodon_transvaalensis
Chloris_elata
Eustachys_distichophylla
Chloris_truncata
Lintonia_nutans
Microchloa_caffra
Harpochloa_falx
Lepturus_repens
Enteropogon_macrostachyus
Trichloris_crinita
Leptochloa_dubia
Astrebla_lappacea
Dinebra_retroflexa
Eleusine_tristachya
Eleusine_coracana
Acrachne_racemosa
Vaseyochloa_multinervosa
Triplasis_americana
Gouinia_latifolia_latifolia
Cleistogenes_songorica
Aeluropus_littoralisAF144597
Schmidtia_pappophoroides
Enneapogon_scoparius
Cottea_pappophoroides
Eragrostis_echinochloidea
Uniola_paniculata
Spartina_pectinata
Zoysia_japonica
Sporobolus_indicus
Centropodia_glauca
Merxmuellera_rangei
clade IV
clade VI
clade I
clade II
clade III
30.0
25.0
20.0
15.0
10.0
5.0
0.0
millions of years before present

Supp. data 3.8

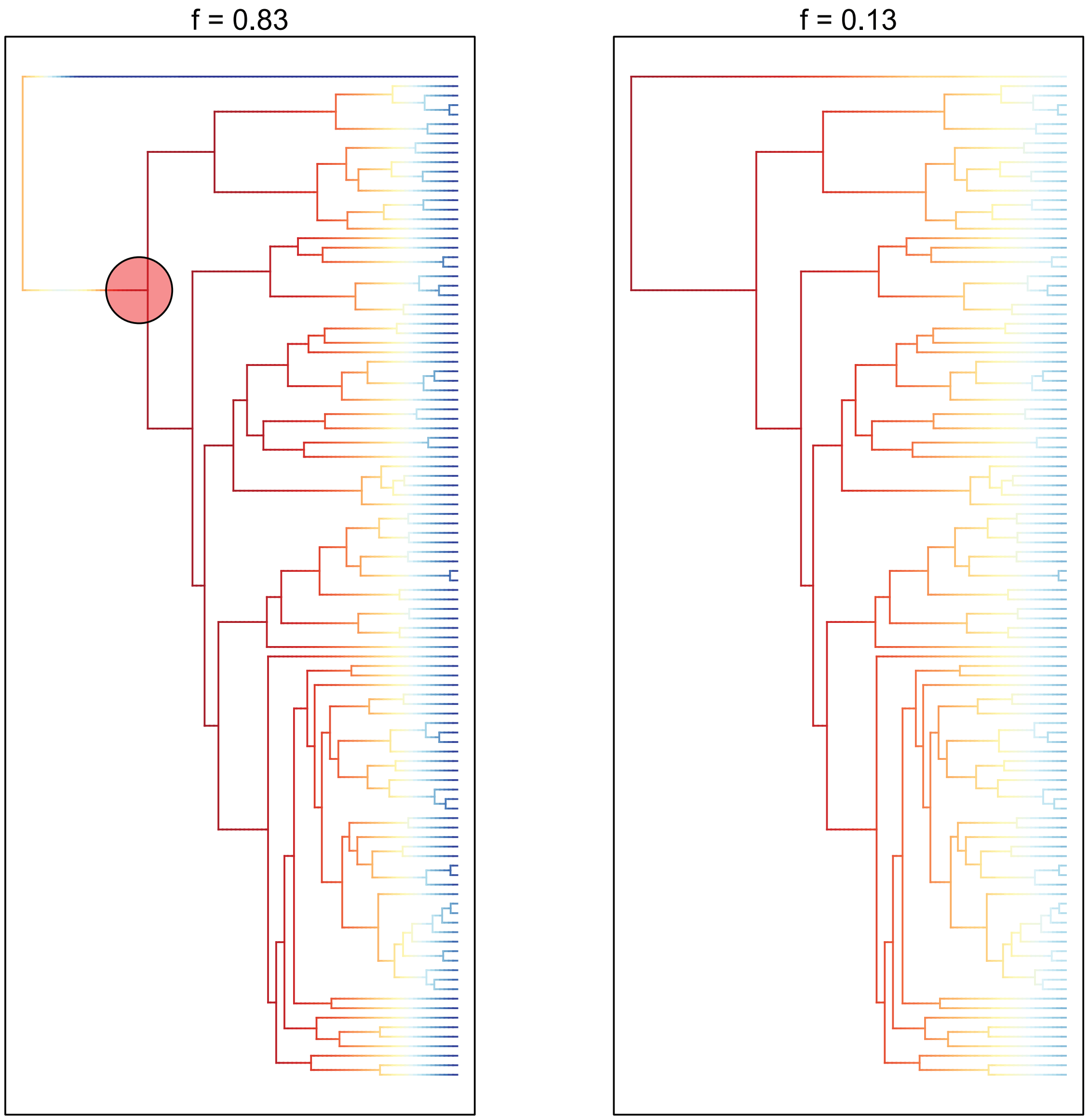

Supp. data 3.9

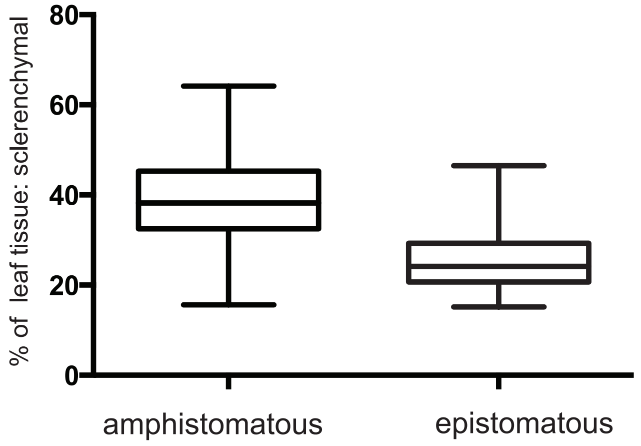
80
60
40
20
0
% of leaf tissue: sclerenchymal
amphistomatous
epistomatous

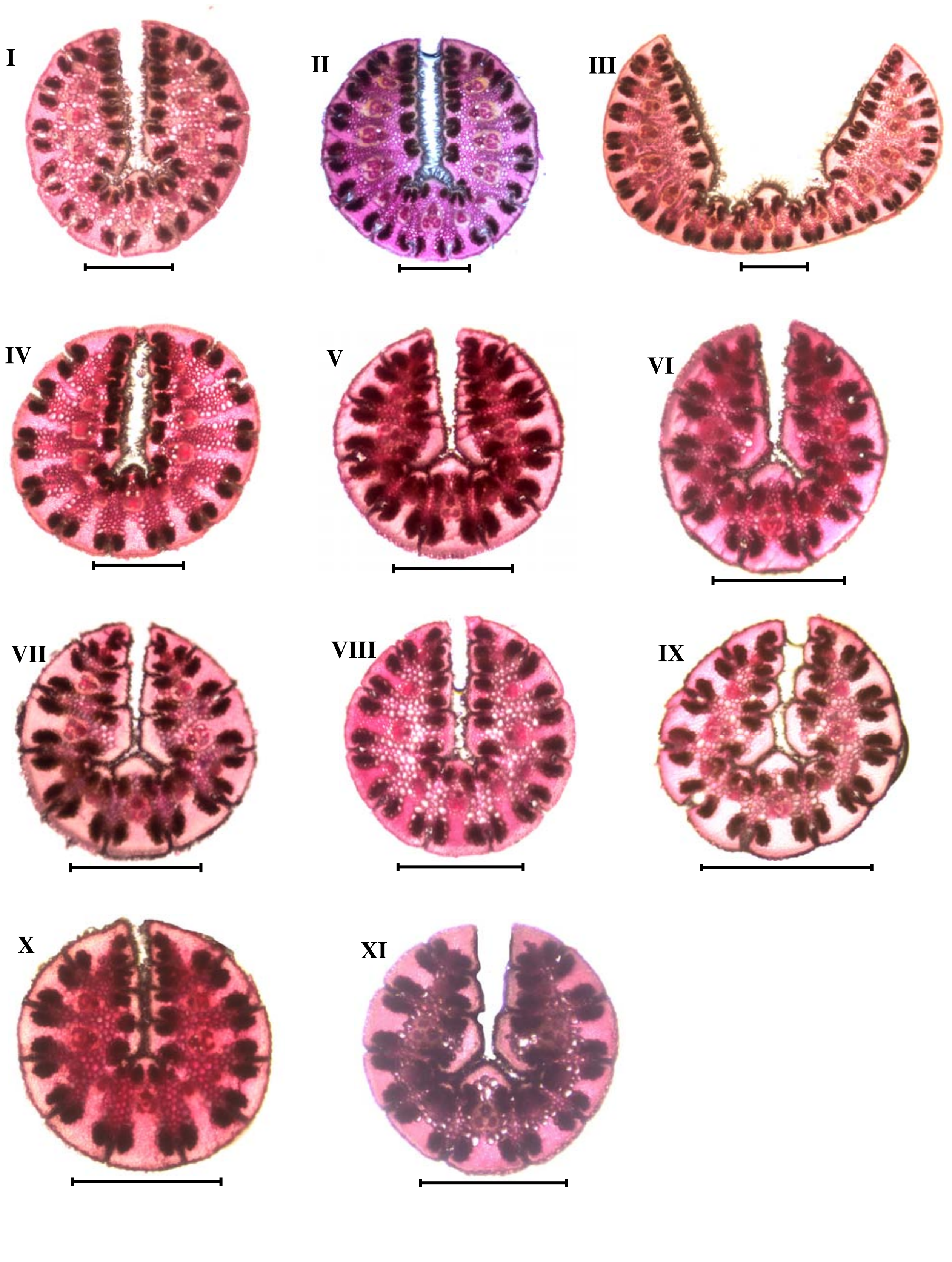

Supp. Data 4. Leaf cross sections of species in clade I and II (Figure 2). I: *T. bromoides*, II: *T. danthonioides*, III: *T. dielsii*, IV: *T. longipalea* (Danthonioides group); V: *T. bunicola*, VI: *T. compacta*, VII: *T. irritans*, VIII: *T. lanata*, IX: *T. scariosa*, X: *T. tomentose*, XI: *T. vella* (Scariosa group). Sections were taken from the middle portion of the leaves. Scale bar = 0.5mm

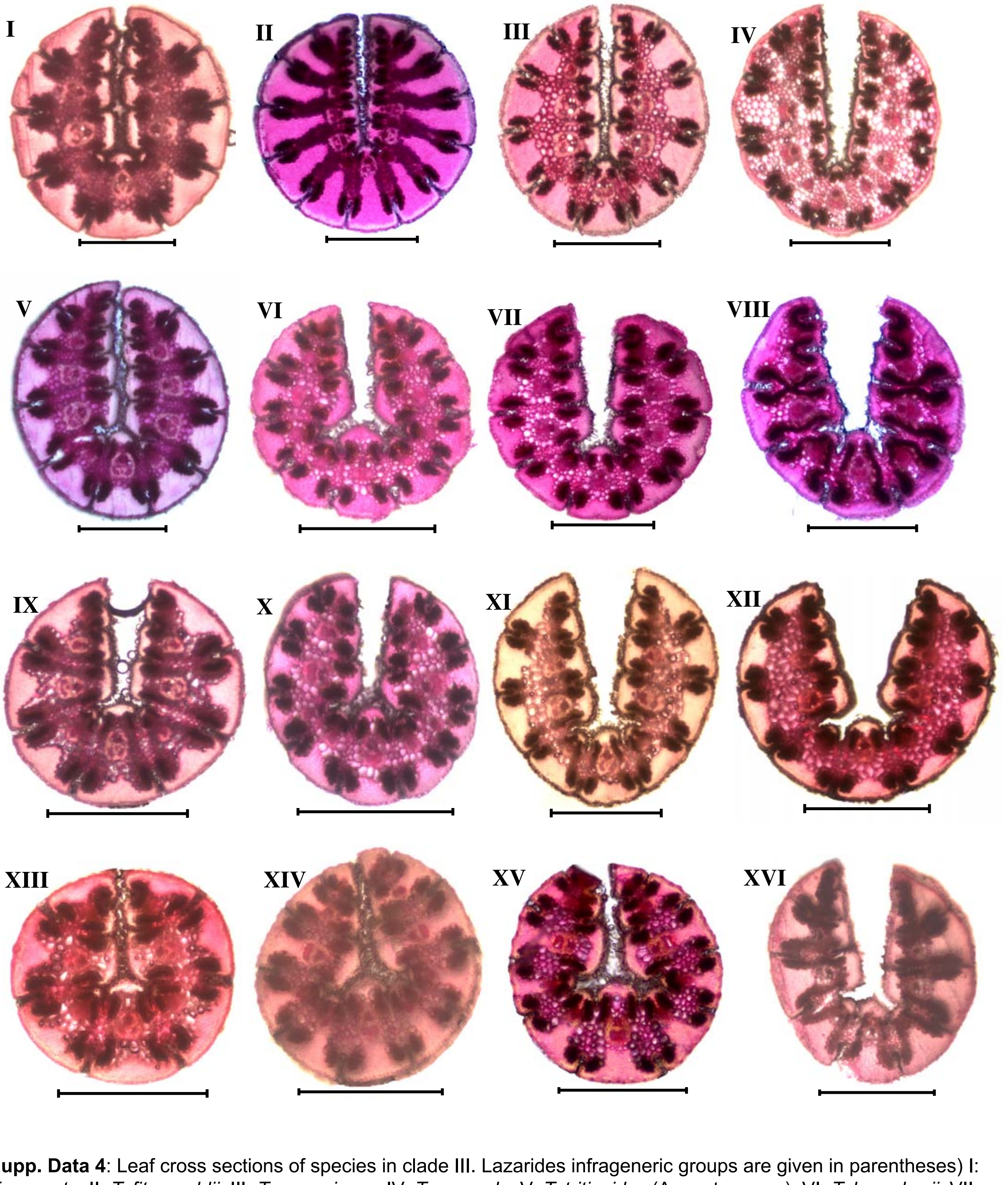

Supp. Data 4: Leaf cross sections of species in clade III. Lazarides infrageneric groups are given in parentheses) I: *T. angusta*, II: *T. fitzgeraldii*, III: *T. racemigera*, IV: *T. secunda*, V: *T. triticoides* (Angusta group); VI: *T. basedowii*, VII: *T. brizoides*, VIII: *T. intermedia*, IX: *T. inutilis*, X: *T. lanigera*, XI: *T. molesta*, XII: *T. roscida*, XIII: *T. wiseana* (Basedowii group); XIV: *T. desertorum*, XV: *T. rigidissima* (Danthonioides group); XVI: *T. aristiglumis* (Plectrachnoides group). Sections were taken from the middle portion of the leaves. Scale bar = 0.5mm

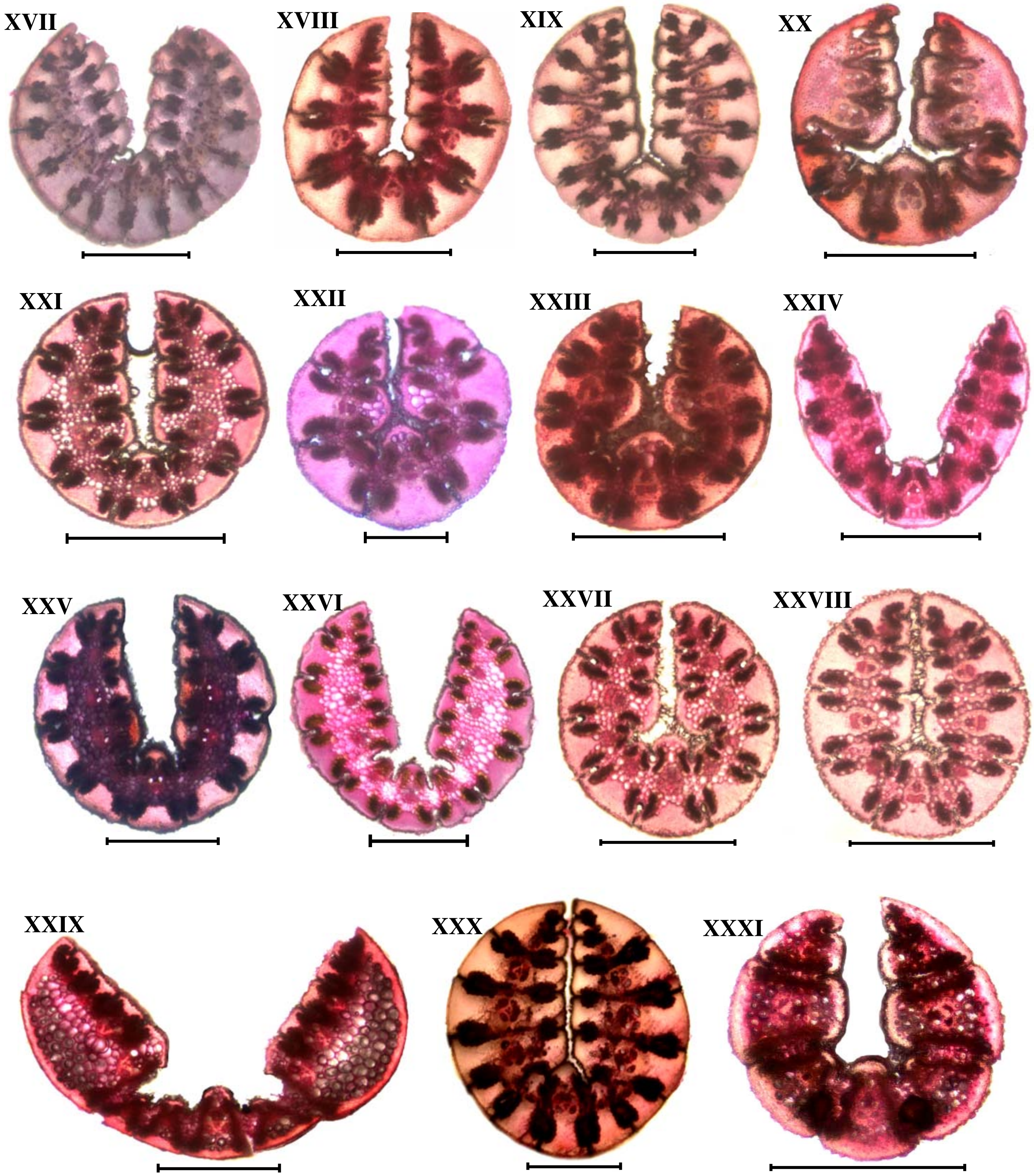

Supp. Data 4: Leaf cross sections of species in clade III continued. XVII: *T. aurita*, XVIII: *T. contorta*, XIX: *T. plectrachnoides*, XX: *T. uniaristata*, (Plectrachnoides group); XXI: *T. pascoeana* (Procera); XXII: *T. aeria* (Schinzii group); XXIII: *T. concinna*, XXIV: *T. inaequiloba*, XXV: *T. integra*, XXVI: *T. longiceps*, XXVII: *T. plurinervata*, XXVIII: *T. spicata*, (Spicata group); XXIX: *Monodia stipoides* (Monodia); XXX: *Symplectrodia lanata*, XXXI: *S. gracilis* (Symplectrodia). Sections were taken from the middle portion of the leaves. Scale bar = 0.5mm

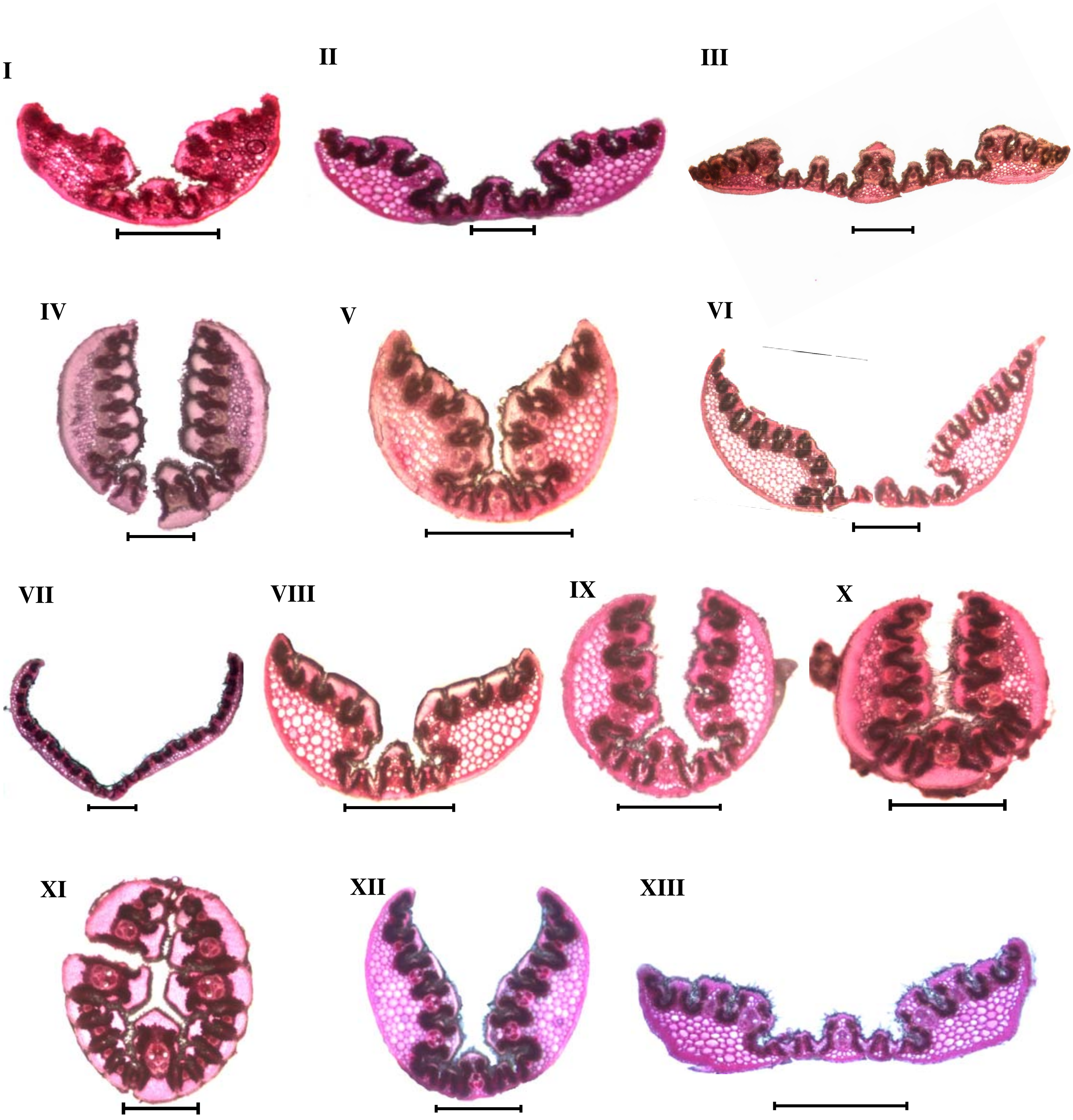

Supp. Data 4.:. Leaf cross sections of species in clade III continued. Lazarides infrageneric groups are given within parentheses. I: *T. biflora*, II: *T. burbidgeana*, III: *T. cunninghamii,* IV: *T. latzii*, V: *T. microstachya*, VI: *T. procera*, VII: *T. radonensis* , VIII: *T. stenostachya*, (Procera group); IX: *T. epactia*, X: *T. hubbardii*, XI: *T. longiloba* , XII: *T. marginata*, XIII: *T. mitchellii* (Pungens group). Sections were taken from the middle portion of the leaves. Scale bar = 0.5mm

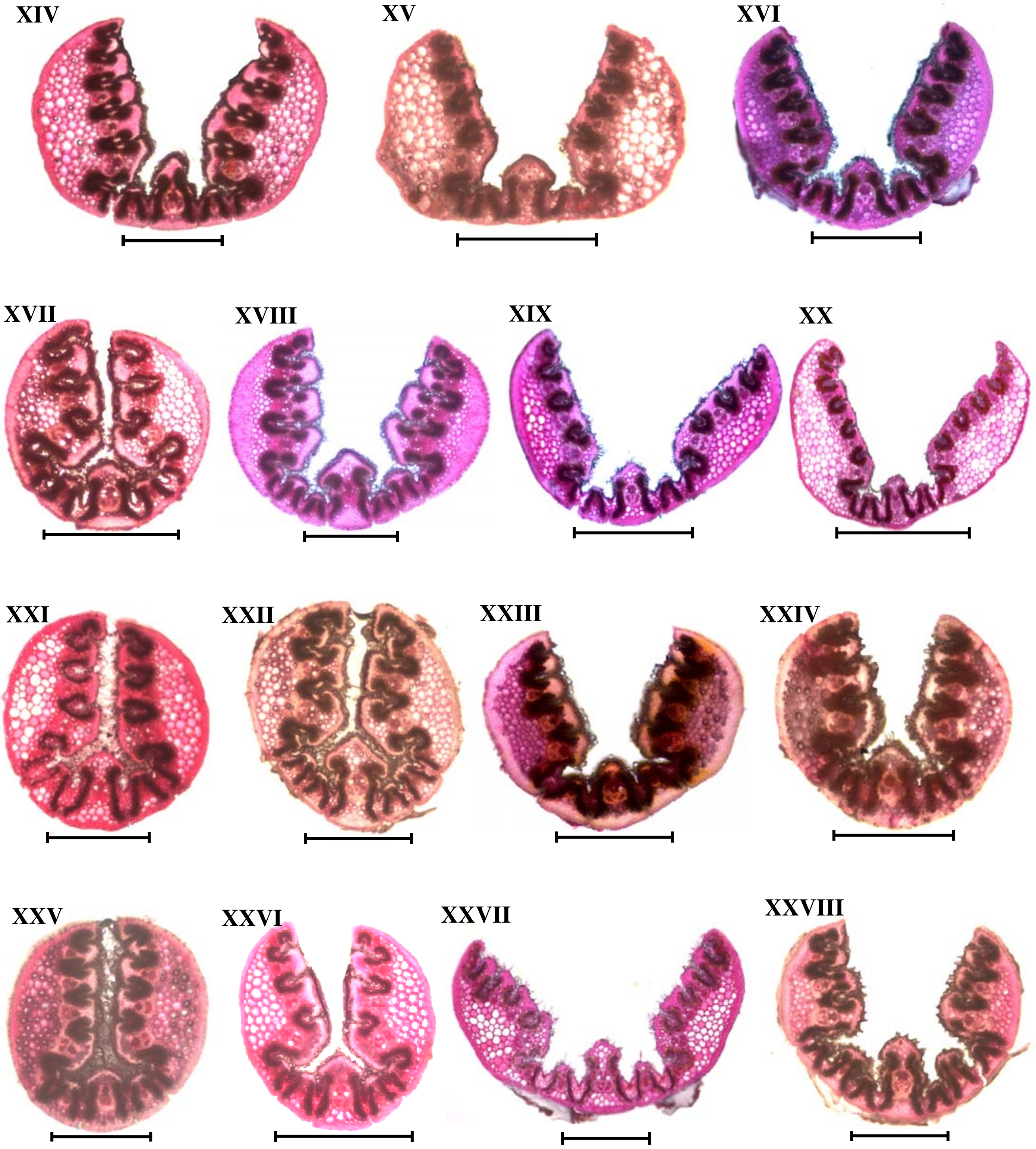

Supp. Data 4: Leaf cross sections of species in clade III continued. XIV: *T. pungens* (Pungens group), XV: *T. acutispicula*, XVI *T. bitextura*, XVII: *T. bunglensis*, XVIII: *T. bynoei*, XIX: *T. caelestialis*, XX: *T. claytonia*, XXI: *T. helmsii,* XXII: *T. melvillei*, XXIII: *T. prona*, XXIV: *T. salina*, XXV: *T. schinzii*, XXVI: *T. sp. aff. claytonia* , XXVII: *T. sp. nov.* (aff.T. schinzii), XXVIII: *T. triaristata* (Schinzii group). Sections were taken from the middle portion of the leaves. Scale bar = 0.5mm
